# Time-resolved core-level photoelectron spectroscopy of glycine fragmentation: a case study on the capabilities and limitations of site-selective probes at XFELs

**DOI:** 10.1039/d6fd00069j

**Published:** 2026-06-17

**Authors:** Antoine Sarracini, Lorenzo Paoloni, Christopher Arrell, Sven Augustin, Loïc Bassement, Christoph Bostedt, Sergio Díaz-Tendero, Davide Faccialá, Zhaoheng Guo, Gregor Knopp, Suddhasattwa Mandal, Ana Sofia Morillo-Candas, Kevin Prince, Zhibin Sun, Hankai Zhang, Xinhua Xie, Antonio Picón, Kirsten Schnorr, Andre Al Haddad

**Affiliations:** a Center for Photon Science, Paul Scherrer Institut 5232 Villigen PSI Switzerland andre.al-haddad@psi.ch; b Departamento de Química, Universidad Autónoma de Madrid 28049 Madrid Spain; c Condensed Matter Physics Center (IFIMAC), Universidad Autónoma de Madrid 28049 Madrid Spain; d Instituto de Ciencia de Materiales de Madrid (ICMM-CSIC) 28049 Madrid Spain; e Center for Scientific Computing, Theory and Data, Paul Scherrer Institut 5232 Villigen PSI Switzerland; f Laboratory for Ultrafast X-ray Sciences, Institute of Chemical Sciences and Engineering, École Polytechnique Fédérale de Lausanne (EPFL) CH-1015 Lausanne Switzerland; g Institute for Advanced Research in Chemical Sciences (IAdChem), Universidad Autónoma de Madrid 28049 Madrid Spain; h CNR, Istituto di Fotonica e Nanotecnologie Piazza Leonardo da Vinci 32 20133 Milan Italy; i Elettra-Sincrotrone Trieste S.C.p.A. I-34149 Basovizza Trieste Italy; j Center for Transformative Science, ShanghaiTech University Shanghai China

## Abstract

This paper presents the time-resolved X-ray photoelectron spectroscopy (TR-XPS) setup at the Swiss Free-Electron Laser (SwissFEL) Maloja endstation for investigating the ultrafast dynamics of gas-phase molecules with site-specificity. As a case study, we investigate the dissociative photoionization of glycine. By tracking the C 1s, N 1s, and O 1s core-level edges, we resolve the site-specific dynamics following multiphoton ionization. Guided by *ab initio* theory, the measurements are modeled using a global kinetic fit. Using this model, we report a glycine parent cation lifetime of 395 ± 25 fs, consistent with the theoretically predicted barrier to C–C bond rupture. We track fragment separation in real time *via* carboxyl red-shifts, which signal the relaxation of coulombic repulsion between the COOH and the iminium ion (CH_2_NH_2_^+^). Finally, we address fundamental technical challenges, including the temporal limitations imposed by laser-induced optical sidebands in the presence of high-intensity fields, understanding the modulated ground-state bleach signal, and the complexities of the theoretical framework that couples *ab initio* molecular dynamics with high-level quantum-chemical calculations of core-level binding energies.

## Abstract 2

Time-resolved X-ray photoelectron spectroscopy captures the site-specific ultrafast fragmentation of the glycine cation, revealing a 400 fs lifetime for C–C bond cleavage.

## Graphical abstract

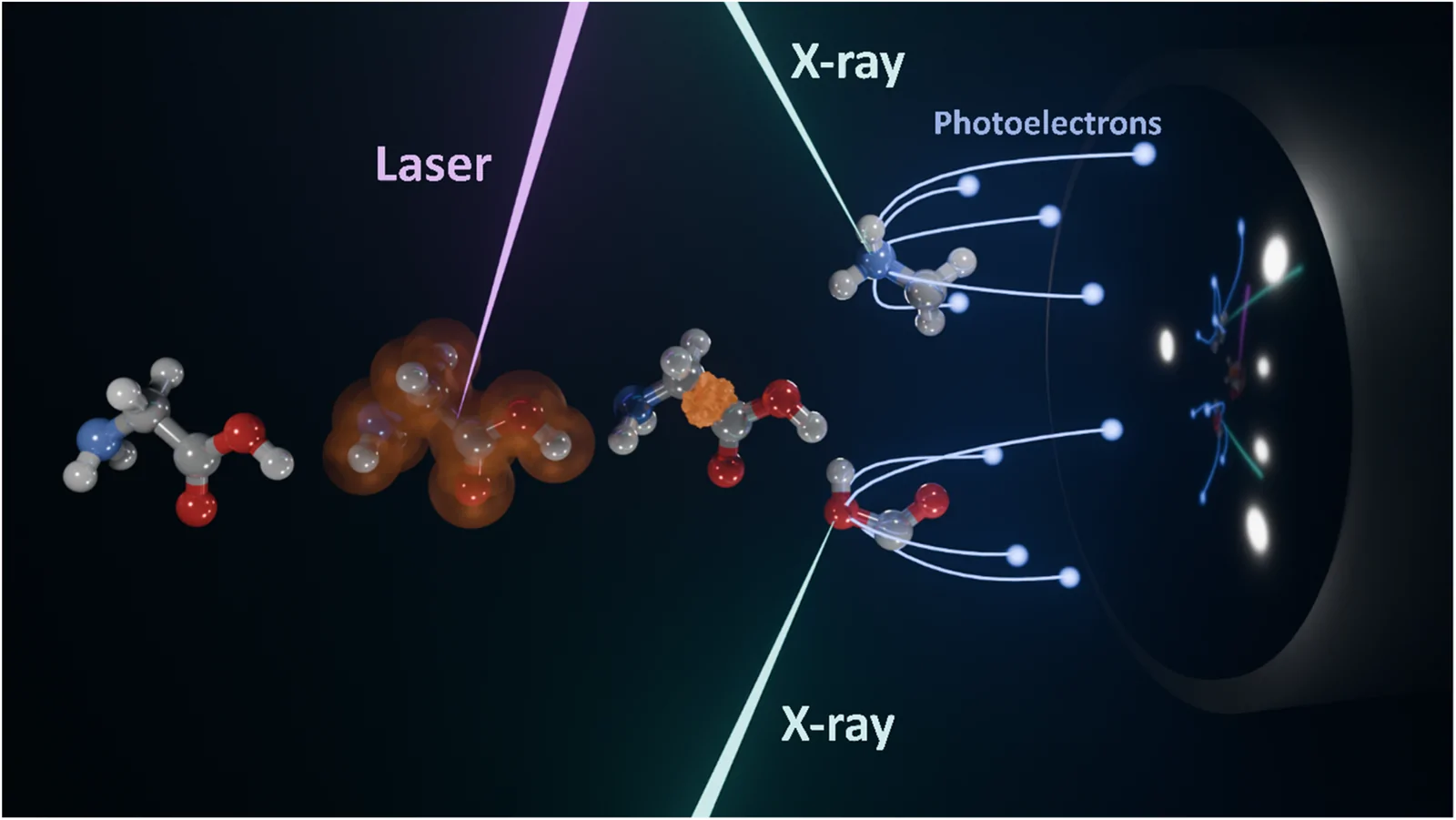

## Introduction

The evolution of X-ray photoelectron spectroscopy (XPS) to the time domain represents a fundamental shift in ultrafast science, allowing the observation of nuclear and electronic dynamics in real time.^1–5^ As a site-specific probe, time-resolved X-ray photoelectron spectroscopy (TR-XPS) enables monitoring of local chemical environments with atomic selectivity.^6,7^ Recent research has established the utility of the core-level shifts as sensitive probes of non-adiabatic dynamics. Picón *et al.* and Inhester *et al.* have provided a theoretical framework demonstrating how TR-XPS tracks, in real time, the chemical environment of a particular element within a molecule.^8,9^ Experimentally, a series of works have demonstrated the use of this technique at X-ray Free Electron Lasers (XFELs) and synchrotrons to resolve site-selective dynamics at the femtosecond^1–5,10–12^ and nanosecond time scale.^13,14^

The investigation of glycine photochemistry has long served as a critical benchmark for understanding the stability and reactivity of one of the simplest biological building blocks. As the smallest naturally occurring amino acid, glycine might appear structurally simple; however, its ionization in the gas phase gives rise to surprisingly rich and non-trivial dynamics. Early theoretical studies of single-photon ionization already showed that the resulting cationic evolution is governed by electronic effects, identifying cleavage of the central C–C bond as the preferred dissociation channel within the first few picoseconds after ionization.^15^ Subsequent experiments using extreme ultraviolet (XUV) and soft X-ray radiation confirmed this picture, revealing ultrafast hydrogen migration competing with dissociation and demonstrating the robustness of the C–C cleavage pathway over a wide range of excitation conditions.^16,17^ More recently, studies employing femtosecond and attosecond X-ray sources have shown that photoionization can prepare glycine cations (Gly^+^) in coherent superpositions of electronic states, whose early-time evolution precedes nuclear motion and strongly influences the resulting fragmentation.^18,19^ Notably, the dominance of the C–C bond rupture is not restricted to photoionization: ion-molecule collisions involving keV and highly charged projectiles, as well as low-energy electron impact, also lead to the same primary dissociation channel in glycine and other simple amino acids, despite the different modes of energy deposition.^20–23^

Beyond fragmentation, these studies have revealed that ionization can also activate further chemical reactivity in amino acid systems. In particular, investigations of doubly ionized species formed in ion-molecule collisions showed that ultrafast intramolecular rearrangements, such as hydrogen and hydroxyl migration, can efficiently compete with Coulomb driven cleavage and substantially modify the molecular structure.^20,24^ Experiments on more complex cyclic dipeptides further demonstrated that vacuum ultraviolet (VUV) photoionization induces systematic ring opening followed by the formation of highly reactive intermediates, thereby linking elementary fragmentation processes to subsequent chemical reactivity.^25,26^ Recent studies have demonstrated that ionization can directly promote peptide bond formation, either following UV/VUV excitation in protonated amino acid clusters or after single ion impact on weakly bound aggregates, leading to the growth of polypeptide chains.^27,28^ These results show that the outcome of amino acid ionization is more diverse than simple fragmentation, and that destructive and constructive pathways may coexist on ultrafast timescales. At the same time, the experimental approaches employed so far (advanced mass spectrometry and coincidence techniques) mainly access global observables such as fragment identities, yields and reaction thresholds. As a consequence, they provide limited information on the time-dependent evolution of the local chemical environment, which is essential for understanding how specific bonds weaken, rearrange and ultimately break. This limitation is particularly relevant for the dominant C–C bond cleavage channel, whose initiation must be traced in site-specific electronic dynamics, motivating the need for a temporally resolved and site-selective probe such as TR-XPS.

While traditional coincidence and mass spectroscopy techniques are highly effective at determining fragment yields and branching ratios, they provide limited insight into the transient electronic environments during dissociation. TR-XPS provides a complementary perspective by enabling the observation of these dynamics through instantaneous probing of the chemical environment of a chosen atomic site. By tracking the C 1s, N 1s, and O 1s edges, we can resolve the evolution of local chemical environments in real time. This approach allows us to monitor the metastability of the Gly^+^ and track the dynamic in binding energy that signals the fragmentation process. A scheme of the experiment showcasing the measurement at the N 1s edge and fragments is shown in Fig. 1. Establishing a synergy between site-specific probes and high-level *ab initio* calculations provides a robust strategy for capturing the electronic rearrangements that govern the fragmentation process.

**Fig. 1:**
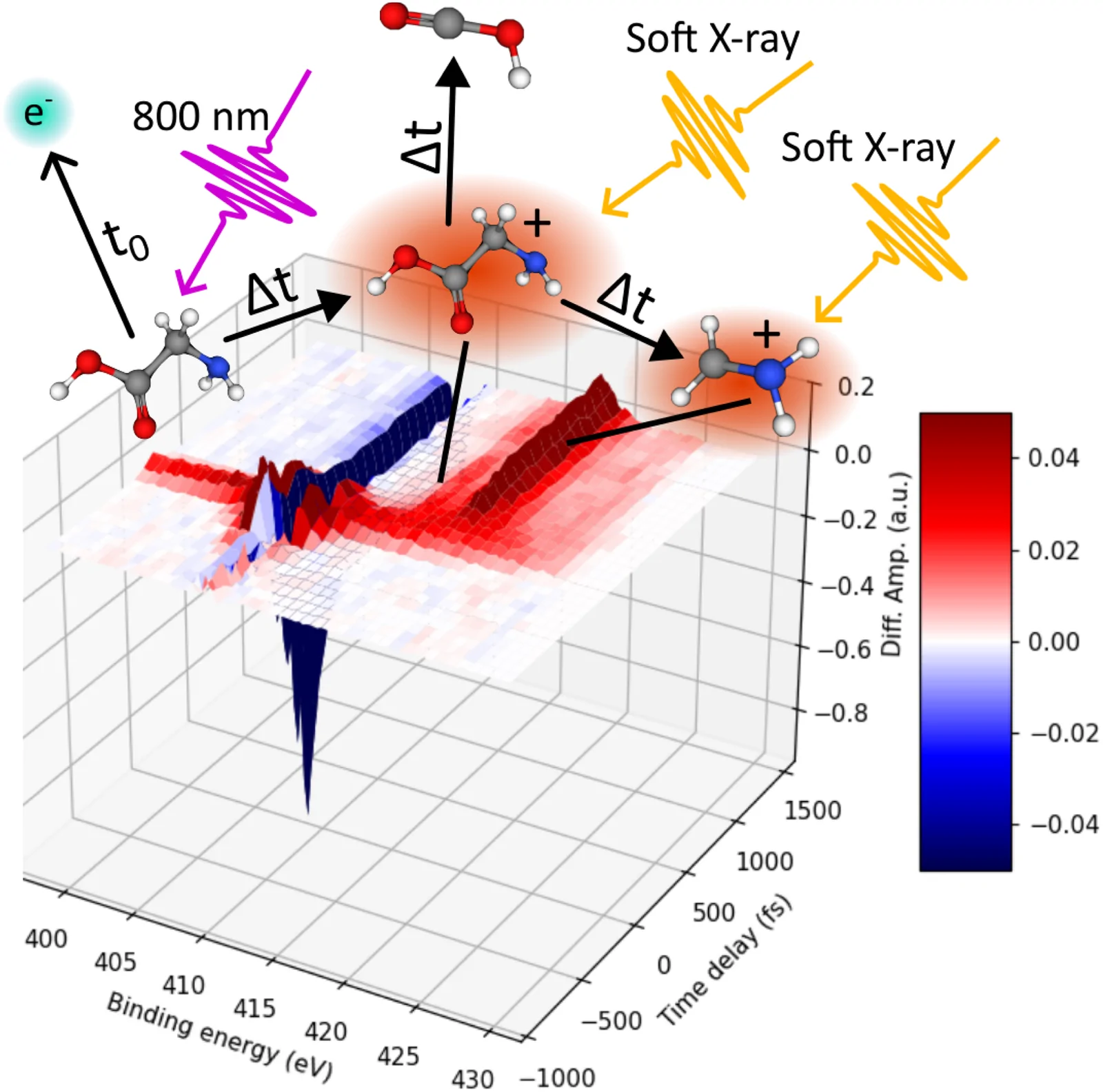
A schematic showing the time-resolved X-ray photoemission experiment performed at the N 1s edge of Gly^+^. The optical laser at 800 nm ionizes glycine molecules in the gas phase; the cation and fragments are probed using soft X-ray pulses.

Utilizing these capabilities at XFELs introduces a fundamental trade-off between spectral and temporal resolution. In standard XFEL operation modes, pulses are often broad-spectrum, leading to spectral smearing in XPS measurements. A monochromator could be employed to achieve high spectral resolution in our setup at the Maloja endstation of SwissFEL, stretching the X-ray pulse temporally. Alternative methods, such as correlation spectroscopy, could be used to overcome this issue. Despite the availability of methods to bypass the spectro-temporal effects introduced by the monochromator, this method could not improve the measurement time resolution beyond the IRF instrument response limit, which includes the laser-X-ray timing jitter. In addition, in the case of multiphoton ionization using an 800 nm laser to photoionize the glycine molecules, the high intensities required lead to the formation of optical sidebands. These signals arise from continuum–continuum coupling and can obscure subtle chemical shifts during the early stages of the reaction (−250–250 fs), complicating data analysis.

In this study, we present the state-of-the-art experimental setup for TR-XPS available at the Maloja endstation at SwissFEL. We provide a technical overview and present glycine photoionization dynamics as a case study. We measure the Gly^+^ lifetime of 385 fs and observe dynamic red-shifts at the carboxyl site, providing direct visualization of the relaxation of coulombic repulsion between developing fragments. To complement the measurements, we use a theoretical framework to simulate the expected TR-XPS response. Using the Atom-Centered Density Matrix Propagation (ADMP) theoretical method, we are able to understand the Gly^+^ lifetime and relate it to the vibrational energy required to overcome the C–C bond rupture barrier. Ultimately, this is achieved while providing a comprehensive assessment of the technique's limitations, particularly regarding the mitigation of strong-field ionization artifacts, such as optical sidebands and space-charge artifacts, and challenges associated with the theoretical methods used.

## Methods

### Experimental method

The experiments were performed at the Maloja endstation, positioned on the Athos branch of the SwissFEL.^29,30^ The Athos beamline is specifically designed to provide high-flux, soft X-ray pulses through its specialized Apple-X undulators, covering an energy range from 250 eV to 1.7 keV.^31^ For the study of glycine fragmentation, the FEL was operated in Self-Amplified Spontaneous Emission (SASE) mode. In this mode, the standard X-ray pulses exhibit a spectral bandwidth of approximately 1% of the central photon energy with a characteristic pulse duration of ∼40 fs (FWHM). To achieve the high spectral resolution necessary to resolve subtle chemical shifts, an inline grating monochromator was employed.^32^ The use of the monochromator reduces the FEL bandwidth to 0.1–0.5 eV, though this spectral refinement inherently stretches the temporal profile of the X-ray pulse.^33^ Pulse stretching is due to the wavefront tilt of the dispersed beam at the exit slit, which is determined by the number of illuminated grating lines and the magnification. As a consequence, the pulse stretch depends on the X-ray central photon energy, ranging from 70 fs at 300 eV to 10 fs at 1.7 keV. The conditions used in this work induced a stretch of around 30 fs in addition to the original X-ray pulse length of 40 fs, yielding an estimated pulse length of 70 fs.

Gas-phase glycine was introduced into the interaction region using a high-temperature effusive oven. Solid glycine was evaporated through a 200 µm diameter orifice, positioned approximately 1 mm below the X-ray beam axis, to ensure a high-density target while maintaining vacuum integrity. Photoelectrons generated by the X-ray probe were detected using a SPECS Phoibos 150 EP hemispherical electron analyzer. This spectrometer was operated in fixed-energy mode, providing high energy resolution with a collection efficiency of approximately 0.5% at 100 eV kinetic energy electrons and a linearly polarized X-ray beam. To enable pulse-to-pulse correlation with the fluctuating FEL parameters, the projection of a single camera frame was recorded for each X-ray shot. This shot-to-shot acquisition strategy enables post-processing correlation of electron kinetic energies with the instantaneous FEL parameters.

The molecular dynamics were initiated using a Ti:Sapphire laser system providing 800 nm pulses with a 30 fs (FWHM) duration. While the Maloja endstation supports a variety of pump wavelengths spanning from ultraviolet to infrared through optical parametric amplifiers and harmonic generation, the fundamental 800 nm pulse was used in this study. The pulses were compressed close to the transform-limited state at the sample interaction. A critical challenge in XFEL pump–probe experiments is the inherent arrival-time jitter between the FEL and the optical laser. To mitigate this, a timing diagnostic based on transient reflectivity in a silicon substrate was positioned downstream of the experimental chamber. This arrival-time monitor (ATM) enables data binning with a temporal resolution of approximately 30 fs.^34^

### TR-XPS of the glycine cation

Time-resolved XPS maps were recorded at the C 1s, N 1s, and O 1s core levels under near-identical experimental conditions. Glycine molecules were ionized *via* a multi-photon process using an 800 nm laser pulse with a pulse energy of 460 µJ and focused to a 150 × 150 µm^2^ spot (FWHM), yielding a peak intensity of 5.6 × 10^13^ W cm^−2^. The X-ray probe beam was focused to 80 × 80 µm^2^, with photon energies tuned to approximately 80 eV above the N 1s and O 1s ionization thresholds, and 120 eV above the C 1s threshold. The X-ray spectral resolution was set to 0.4 eV. TR-XPS maps were acquired over a 1.5 ps temporal window with an initial step size of 40 fs, as shown in the schematic in Fig. 1.

The data processing was limited to normalizing individual XPS spectra to the FEL pulse energy (*I*_0_) and correcting for arrival-time jitter using a timing diagnostic. Given that the laser intensity fluctuations remained below 1%, no additional laser power correction was required. After jitter correction, the data were rebinned along the time axis into 10 fs steps. Spectra recorded before time zero were then subtracted from the maps to extract the underlying transient dynamics. The differential maps of each element are presented in Fig. 2a–c.

**Fig. 2:**
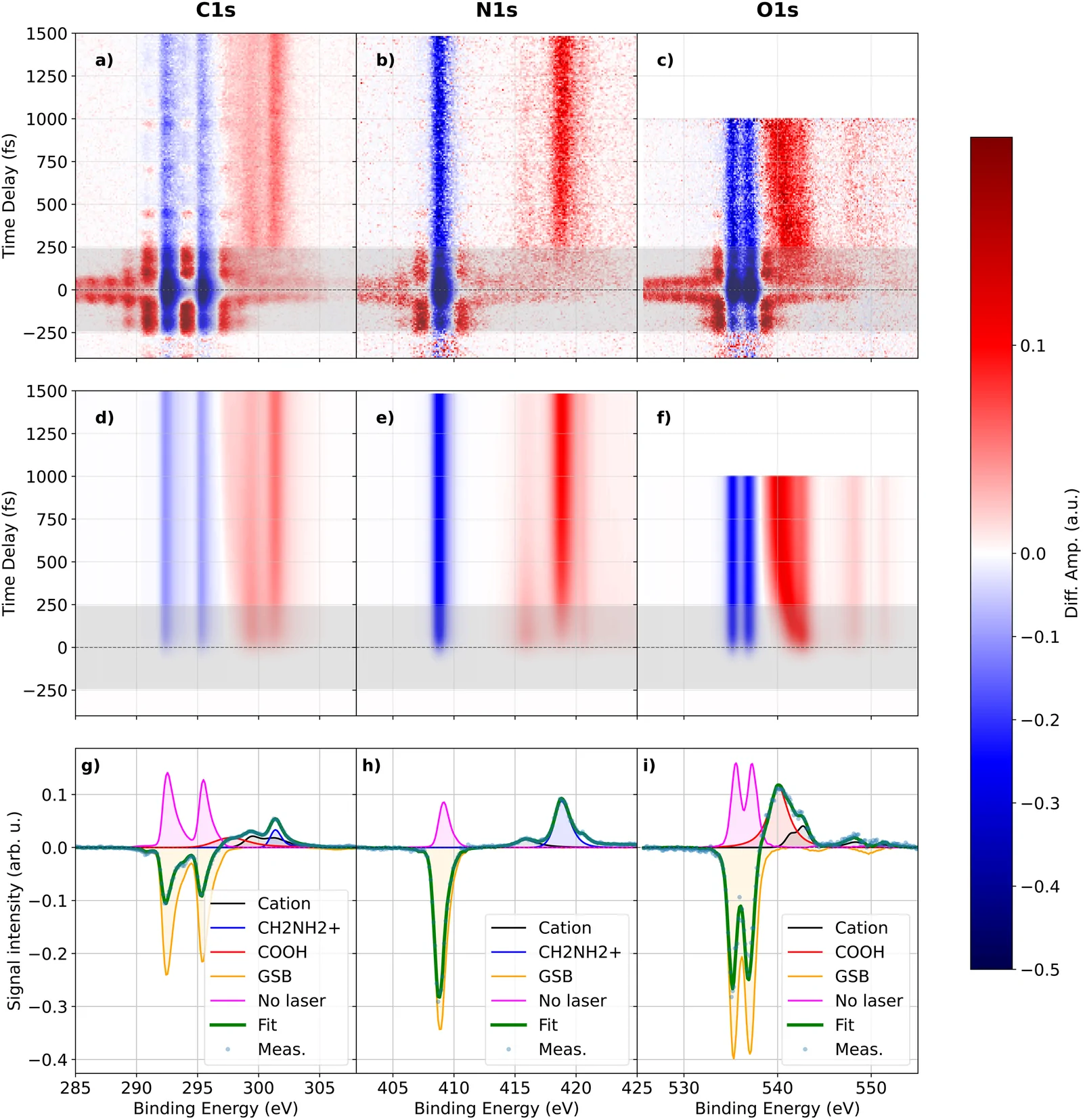
(a–c) Transient XPS maps of Gly^+^ at the C 1s, N 1s, and O 1s edges. (d–f) Global multi-exponential fitting model to describe the measurements. (g–i) Measurement (blue circles) overlapped with the model (green) at 750 ± 50 fs, with the individual species spectra.

### Modeling

To extract the transient dynamics from the experimental maps, a global time-dependent fitting routine is used. The spectral landscapes are modeled as a set of photoemission lines whose intensities, positions, and widths are allowed to evolve along a temporal coordinate, the pump–probe delay. For each state or fragment, a series of lines are used with independent amplitudes, energies, and line shapes, ultimately scaled by a concentration factor to fit the data.

The photolines are modeled using asymmetric line shapes to account for the physical characteristics of the photoemission process. Each photoline *I*(*E*) at a given kinetic energy *E* is defined as a linear combination of Gaussian (*G*) and Lorentzian (*L*) functions, modified by a mixing parameter *η* and a skewness factor to account for the asymmetric tailing:^35^1
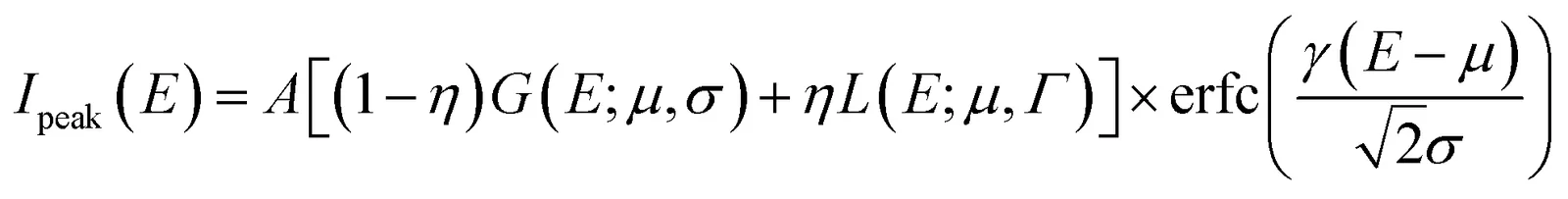
where *A* is the amplitude, *µ* is the central binding energy, *γ* is the skew parameter, *Γ* is the FWHM of the Lorentzian lineshape and *σ* is the width of the spectral resolution of the experiment.

The transient response of the system following excitation is captured by allowing the peak parameters to evolve with the pump–probe delay *t* through exponential decay/rise. To account for time-dependent vibrational broadening and coulombic repulsion between the fragments, the central energy *µ*(*t*) and the line width *W*(*t*) (FWHM) follow exponential evolution:

2 *µ*(*t*) = *µ*_static_ + Δ*µ*(1 − e^−(*t*−*t*_0_)/*τ*_*µ*_^)

3 *W*(*t*) = *W*_static_ + Δ*W*(1 − e^−(*t*−*t*_0_)/*τ*_W_^)

The intensity *A*(*t*) of the species is modeled using multi-exponential kinetics to describe the population dynamics of specific chemical states.

The global optimization is performed by minimizing the cost function 
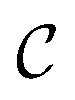
, which extends the standard *χ*^2^ method with two regularization terms. These terms enforce physical boundary conditions on the spectral evolution:4
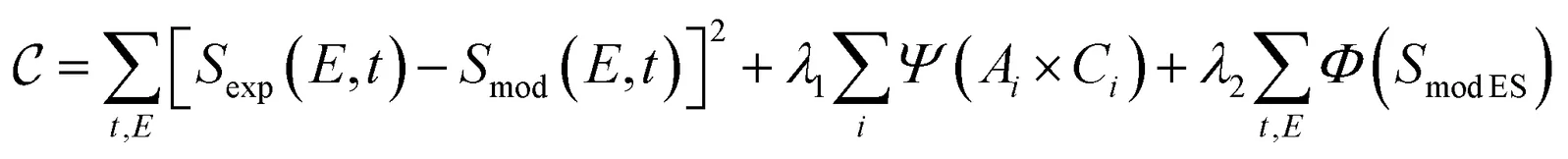
where *S*_exp_ and *S*_mod_ are the measured and modeled difference maps, respectively. The first penalty term, scaled by *λ*_1_, ensures reasonable amplitudes of the individual states with minimum total intensity. The function *Ψ* adds a penalty on growing amplitudes *A*_*i*_ and concentrations *C*_*i*_ of individual species and prevents the appearance of competing features with unrealistically large ground-state bleach (GSB), which ultimately cancel each other out.

The second penalty term, scaled by *λ*_2_, ensures that the populations of the excited states remain positive. While the differential experimental signal shows negative features due to ground-state depletion, the physical intensity of the excited states must remain positive. The function *Φ* penalizes any instances where the modeled transient excited states *S*_mod ES_ are negative:5
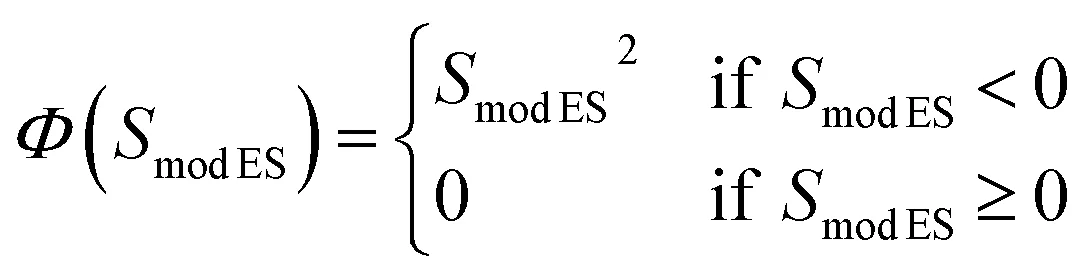


By tuning these hyperparameters, we ensure positivity of the excited states and minimize the total population amplitudes, limiting the overestimation of the initially excited population, hence maintaining a coherent stoichiometry across the transient map.

The optimization was done in a two-step process, starting with 100 000 iterations of global stochastic optimization using the Nelder–Mead method to search for a global minimum of the model. The parameters were started using physical parameters and guidance from the theory, as will be described in the Results section. Due to the high dimensionality and correlated parameters, standard uncertainty estimates are unreliable. In the second step, confidence intervals were evaluated only for key physical parameters, including spectral width, amplitudes, and lifetimes. To ensure these estimates were robust, the parameter boundaries were kept sufficiently broad while remaining within physically meaningful limits. Details on the procedures, initialization, and residuals of the fits are provided in SI Section S2.

Because the model cannot account for the sidebands, the time delays between −250 and 250 fs (grey rectangle in Fig. 2) are excluded from the fitting procedure. The fitted models for each of the elements are presented in Fig. 2d–f. The quality of the fit is assessed by taking spectra and temporal cuts at selected energies and time scales, and comparing the model and measurements. In Fig. 2g–i we present the spectrum integrated over a region of 750 ± 50 fs (blue circles) overlapped with the model (green). The individual species spectrum, GSB, Gly^+^, and individual fragments are presented in shaded colors to highlight their positions and contributions to the fit. Transient cuts are presented in Fig. 4. The description of the number of species and photolines, as well as their dynamics, is detailed in the “C1s transient dynamics and cation evolution” and “O1s transient dynamics and cation evolution” sections.

### Computational details

The computational strategy adopted in this work is designed to support the interpretation of the time-resolved X-ray photoelectron spectroscopy measurements by combining molecular dynamics simulations with high-level quantum-chemical calculations of site-specific core-level energies. Rather than relying on a single theoretical approach, a multistep framework is employed to capture both the structural evolution of ionized glycine following strong-field ionization and the corresponding changes in its electronic structure. In particular, the simulations aim to single out the dominant fragmentation pathways and to identify the evolution of core-level binding energies at chemically distinct atomic sites along the fragmentation process, enabling a direct comparison with the experimental measurements.

The fragmentation dynamics of the Gly^+^ were investigated using the Atom-Centered Density Matrix Propagation (ADMP) molecular dynamics approach,^36–38^ as implemented in the Gaussian16 suite of programs.^39^ In this method, nuclear motion is described by classical trajectories, while the electronic degrees of freedom are propagated adiabatically through an extended-Lagrangian formulation of the density-matrix dynamics. This framework enables the simulation of bond breaking and intramolecular rearrangements in molecular systems described with atom-centered basis sets and has been successfully applied to problems involving ultrafast molecular fragmentation processes.^40–47^

In the present study, ADMP simulations were employed to identify the main intermediates and fragmentation products formed following strong-field ionization of glycine, which results in the formation of an internally excited Gly^+^ cation. The simulations were carried out at the CAM-B3LYP/6-31++G(d,p) level of theory. A time step of 0.1 fs was used together with a fictitious electronic mass of 0.1 amu, thus ensuring adiabaticity during nuclear motion. Molecular dynamics trajectories were propagated up to a maximum simulation time of 300 fs.

Initial conditions for the nuclear dynamics were set with small random nuclear displacements (and random velocities) starting from the equilibrium geometries of neutral glycine. The second lowest-energy conformer of neutral glycine was used as the initial structure, for reasons that will be clarified later. In our study, we took into account the five lowest-energy conformers of neutral glycine (see SI Section S3),^48–50^ with special attention to the two lowest energy conformers identified in high-resolution rotational spectroscopy studies.^51^ To mimic the internal excitation imparted by strong-field ionization, initial nuclear kinetic energies of 0.5, 1, 2, and 5 eV were assigned. In this strong-field ionization regime, it is assumed that the excess electronic excitation energy is rapidly transferred to nuclear degrees of freedom; consequently, the molecular dynamics simulations were performed on the ground electronic state of the ionized molecule. For each energy value, 20 independent trajectories were propagated, providing a statistical sampling of the dominant fragmentation pathways explored by Gly^+^.

Transient XPS signals resulting from the fragmentation of the Gly^+^ were simulated through quantum chemical calculations. Specifically, post-Hartree–Fock calculations were carried out for different nuclear configurations. For each configuration, XPS signals were explicitly calculated by combining:

• Second-order perturbative corrections on top of Complete Active Space Self-Consistent Field calculations (CASPT2 on top of CASSCF) to calculate electronic wavefunctions and energy levels of the Gly^+^;

• Second-order perturbative corrections on top of Restricted Active Space Self-Consistent Field calculations (CASPT2 on top of RASSCF) to calculate electronic wavefunctions and energy levels of the core-ionized Gly^+^;

• Dyson amplitudes between the ground electronic state of the Gly^+^ and its core-ionized electronic states.

### Active space and orbital optimization

XPS calculations were performed with the OpenMolcas software package using the ANO-S basis set.^52^ The Gly^+^ system comprises 39 electrons. The molecular orbital (MO) space was partitioned into 16 inactive orbitals (doubly occupied in all configurations) and 7 active orbitals (in which all possible occupations are allowed). A doublet spin state was assumed, and the optimization of the MOs was performed for the average energy of 10 electronic states.

To access the core-ionized states of Gly^+^, a singly-occupied orbital is enforced in a 1s orbital, thereby creating a core hole. The hole is created in one of the five core shells of interest: the two carbon atoms, the two oxygen atoms, or the nitrogen atom of Gly^+^. Subsequently, 30 of the 38 electrons pertaining to the system (which is now a dication) were placed in 15 inactive orbitals, one in the singly occupied 1s orbital containing the hole, and the remaining 7 in a space of 7 active orbitals. A singlet spin state was assumed, and the MOs were optimized minimizing the average energy of 10 electronic states.

## Results and discussion

The results of the TR-XPS measurements are presented for each of the element edges, C 1s, N 1s, and O 1s. For each element, we will discuss how the model was initialized and how each element/fragment was assigned. The lifetimes of the Gly^+^*τ*_cat_ were treated as a global parameter and fitted across all models to enforce fidelity. In Fig. 2, we present the measured transient map, model, and decay-associated spectra. In Fig. 3, we compare the extracted spectra with theory. Finally, in Fig. 4, we present transients taken from measurements and the model for comparison. After that, we discuss the results in light of the theoretical model to understand the underlying effect driving C–C bond cleavage and the observed lifetime.

**Fig. 3:**
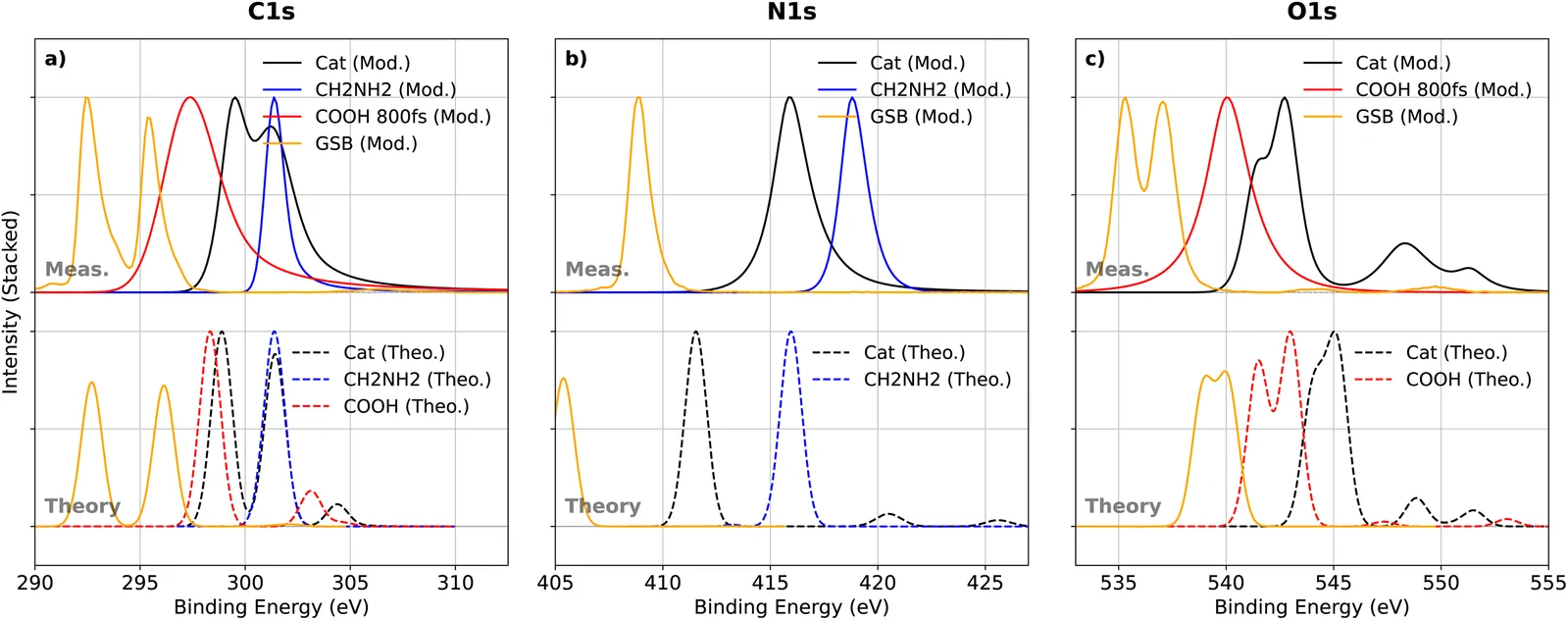
Results from the global fitting and theoretical calculations at (a) C 1s, (b) N 1s and (c) O 1s sites. The top row shows results of the global kinetic model for the Gly^+^ and individual fragments after dynamical shifting. The bottom row shows theoretical spectra for each chemical species are indicated using the same colors as for the measurements, but with dashed curves. Theoretical XPS spectra of Gly^+^ were calculated at the equilibrium geometry of conformer I of neutral glycine.

**Fig. 4:**
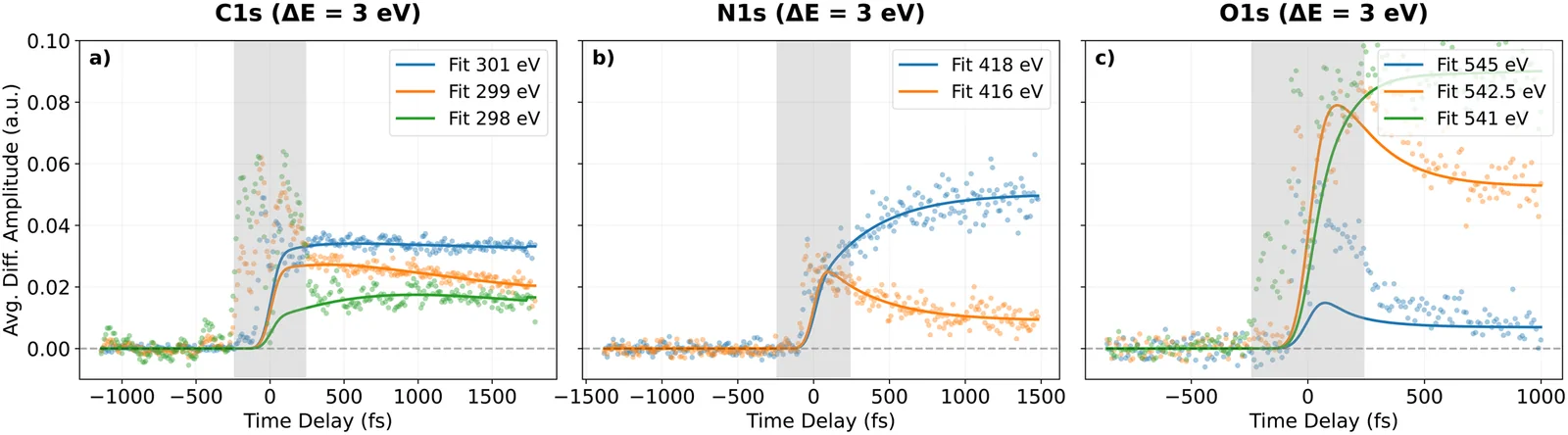
Transient cuts of (a) C 1s, (b) N 1s and (c) O 1s difference maps at selected binding energies at the peak positions of the excited state and ground-state bleach signals. The solid lines represent the model, while the dots are from the measurements.

### C 1s transient dynamics and cation evolution

We start with the calculated C 1s spectra of the Gly^+^, which exhibits two distinct peaks at 298 eV and 301 eV, as presented in Fig. 3a. For ease of discussion, a site-specific assignment is made for each carbon atom: the carboxyl carbon (C_carb_) and the α-carbon (C_α_). The cation spectrum, shown in dashed black, shows two peaks, one for each carbon atom. Upon fragmentation, which is modeled at a C–C bond separation of 8 Å, the C_α_ photoline maintains its position at 301 eV. In contrast, the C_carb_ photoline shifts toward lower binding energies. This shift is not static but increases dynamically as the fragments separate, driven by the decreasing coulombic repulsion from the positively charged iminium (CH_2_NH_2_^+^) fragment as the distance between the two charged centers increases. The dynamic shift due to Coulomb repulsion has been reported previously by Rivas *et al.* for fluoromethane.^4^ This shift is attributed to the increased effective nuclear charge and modified core-hole shielding of the final state within the positively charged iminium moiety.

The experimental C 1s transient map in Fig. 2a shows two distinct excited-state photolines centered at 299 eV and 301 eV, accompanied by a weaker red-shifting feature. Based on theoretical assignments, the two photolines correspond to the initial glycine Gly^+^ prior to its fragmentation. Temporal traces of these lines (Fig. 4a) reveal a non-exponential decay of the 299 eV feature, while the 301 eV line exhibits a concomitant rise and decay. This behavior suggests that the 301 eV signal involves an overlap between the Gly^+^ and a subsequent CH_2_NH_2_^+^ fragment photoline. The fragment photoline at 301 eV can hence be assigned to C_α_. The non-exponential decay of C_carb_ could be described by the appearance of the COOH fragment at the same binding energy, but then red shifting over time.

Equipped with this information, we can now prepare the multi-exponential model. The cation species was modeled by two photolines at 299 and 301 eV, with photon energy, width, asymmetry, and decay rate as fit parameters. To model the dissociation into the fragments, two additional species were introduced for the C_carb_ and C_α_ sites, respectively. The initial binding energies were set to 298 eV (C_carb_) and 301 eV (C_α_). The C_α_ photoline was energetically fixed throughout the fragmentation process, consistent with the localization of the positive charge on the amino moiety.

In contrast, the C_carb_ photoline was modeled with time-dependent parameters for both its central energy and spectral width, following the exponential evolution described by eqn (2) and (3). The dynamic red-shift ranged from 0 eV to −5 eV to account for the evolving electrostatic environment as the neutral carboxyl radical separates from the positively charged iminium partner. The resulting fit, presented in Fig. 2d, allows for the extraction of species-specific spectra and temporal transients with a model integration window of ±1.5 eV (Fig. 4a).

The optimized spectral profiles for the cation and the fragments are presented in Fig. 3a. The cationic spectrum exhibits significant broadening relative to the measured ground state, a signature of the highly vibrationally excited state following multiphoton ionization. The iminium fragment peak (blue) overlaps with the 301 eV cationic signal, explaining the temporal discrepancy observed between the 299 eV and 301 eV decay transients. Meanwhile, the C_carb_ photoline (red) undergoes a dynamic red-shift by 3 eV over a 1.66 ps timescale. Based on the C 1s dataset alone, the ion is found to decay into the fragments with a time constant of around 395 ± 60 fs.

### N 1s transient dynamics and cation evolution

The calculated Gly^+^ photoline appears at 411 eV, as shown in Fig. 3b. Following the rupture of the C–C bond, the nitrogen peak of the CH_2_NH_2_^+^ fragment is shifted further toward higher binding energy (419 eV).

The N 1s transient data exhibit a high degree of clarity due to the presence of a single nitrogen atom in the glycine molecule. As shown in the experimental transient maps (Fig. 2b), two primary excited-state photolines are observed at 416 eV and 419 eV. Although our calculations confirm the presence of two photolines, the predicted energies do not match the experimental results: our calculated data are red-shifted for all the species (neutral glycine, Gly^+^ and the CH_2_NH_2_^+^ fragment). A reason that could explain this substantial offset (not observed at carbon and oxygen K-edges) is that it is difficult to simulate the final core-ionized state, especially in the case of Gly^+^ and the CH_2_NH_2_^+^ fragment. Our calculations suggest that the final core-ionized state in nitrogen could be a triplet, which may explain the mismatch in binding energies between the experimental and calculated N 1s spectra. However, based on the trend of the chemical shift relative to the calculation, we can assign the 416 eV feature to the initial Gly^+^. The 419 eV feature represents the subsequent formation of the iminium fragment.

Transient traces at these binding energies (Fig. 4b) demonstrate a clear kinetic correlation, with the decay of the 416 eV cationic peak mirrored by a rise of the 419 eV fragment signal. The global fitting procedure was defined as a two-species model, where each species was represented by a single photoline. The cation-to-fragment transition was defined by the global time constant, which converged to (395 ± 60) fs, similar to the C 1s. In addition, the pre-*t*_0_ and no-laser spectra were added to the model, similar to the C 1s case.

Because the N 1s photolines for the cation and the fragment are energetically well-separated and remain stationary throughout the dissociation process, the N 1s edge serves as the most reliable reporter for the Gly^+^ lifetime. Furthermore, the optimized spectral width of the cationic photoline was found to be 2.05 eV (FWHM), representing a significant broadening compared to the pre-*t*_0_ ground-state width of 1.14 eV. This observation provides further evidence of a vibrationally “hot” cation resulting from the initial multiphoton ionization process.

### O 1s transient dynamics and cation evolution

Finally, the O 1s spectrum of the cation is centered at 544.5 eV and consists of two peaks, corresponding to carbonyl and hydroxyl oxygen atoms. Upon dissociation, the oxygen fragment photolines shift toward lower binding energies and exhibit a wider peak separation. As with the carbon case, this shift toward lower binding energy is a dynamic process that scales with the C–C bond separation. The shift reflects the relaxation of the potential energy surface as the fragments reach the asymptotic limit.

The O 1s region represents the most congested dataset due to the high density of energetically overlapping photolines. To model these dynamics, the cationic species was represented by two primary photolines and two high-binding-energy shake up satellite peaks. The initial splitting of the primary lines was initiated to match the ground-state spectrum but shifted toward higher binding energies, as predicted by the theoretical model and observed in the transient map. This placed the main cationic features at approximately 544 eV, in agreement with the experimental transient map.

The COOH fragment was modeled using a lineshape similar to that of the cation, but initiated at 542 eV. These features were allowed to red-shift toward lower binding energies to account for the evolving coulombic repulsion as the fragments separate. The final fit, presented in Fig. 3c, shows that the cation spectrum is in excellent agreement with the theoretical prediction, despite a minor energy offset. In contrast, the modeled COOH signal exhibits a single peak rather than the expected doublet and possesses a non-ideal lineshape. This discrepancy is likely due to high spectral congestion, which limits the model's ability to accurately resolve the COOH contribution.

While the O 1s data provides qualitative confirmation of the red-shift and temporal decay behavior observed in the Gly^+^, it is the least reliable dataset for extracting detailed structural information.

### Theory and measurements unite: disentangling the fragmentation mechanism

The alignment between our measurements and theoretical predictions allows us to interpret the (395 ± 25) fs Gly^+^ lifetime as a definitive measure of the C–C bond-rupture mechanism; in this section, we will explain this timescale by relating it to the excess vibrational energy present in the Gly^+^.

Multiphoton ionization removes an electron from the nitrogen lone pair, the highest occupied molecular orbital (HOMO) of the molecule. This localization induces immediate electronic stress on the C–C backbone, weakening the bond and lowering the dissociation activation barrier. As established in the seminal work of Díaz-Tendero *et al.*,^20^ the C–C bond is the most prone to breaking because single ionization of the nitrogen lone pair specifically weakens this central coordinate, and the resulting dissociative channel is the lowest-energy path available to the cation. The preference for this coordinate is driven by the positive charge being preferentially retained on the iminium fragment, which has a lower ionization potential. Hence, this yields the dominant production of CH_2_NH_2_^+^ and a neutral COOH radical.

Our TR-XPS data provides a real-time window into the Gly^+^'s metastability. While traditional mass spectrometry and coincidence techniques are highly effective at determining asymptotic fragment yields, they remain blind to the transient electronic phases of the reaction. From our data, we observe the metastable cation and significant spectral broadening of the photolines, as seen in the N 1s spectrum, with a broadening of 0.91 eV relative to the ground state. This confirms the creation of a vibrationally “hot” cation that must redistribute its internal energy to overcome a dissociation barrier, explaining why fragmentation is not instantaneous. Theoretical input is essential to understand this barrier and its impact on the Gly^+^'s lifetime.

To support this interpretation without the computational demand of a full *ab initio* treatment, we adopted an alternative strategy using ADMP molecular dynamics. In this approach, we deposit a controlled amount of nuclear kinetic energy (0.5–5 eV) randomly to approximate the rapid redistribution of post-ionization energy among nuclear modes. Results from 80 ADMP trajectories (Fig. 5) demonstrate that C–C bond rupture is highly sensitive to the deposited energy. At lower energy levels (0.5–1 eV), dissociation is less frequent and occurs on timescales up to several hundred femtoseconds. At higher energies, the dissociation pathways increase drastically, speeding up the time scales to 100–200 fs. This provides strong evidence for the small energy barrier to fragmentation. These trajectories were propagated from initial geometries based on the second neutral glycine conformer. However, all five conformers are experimentally accessible due to the thermal conditions of sample vaporization.

**Fig. 5:**
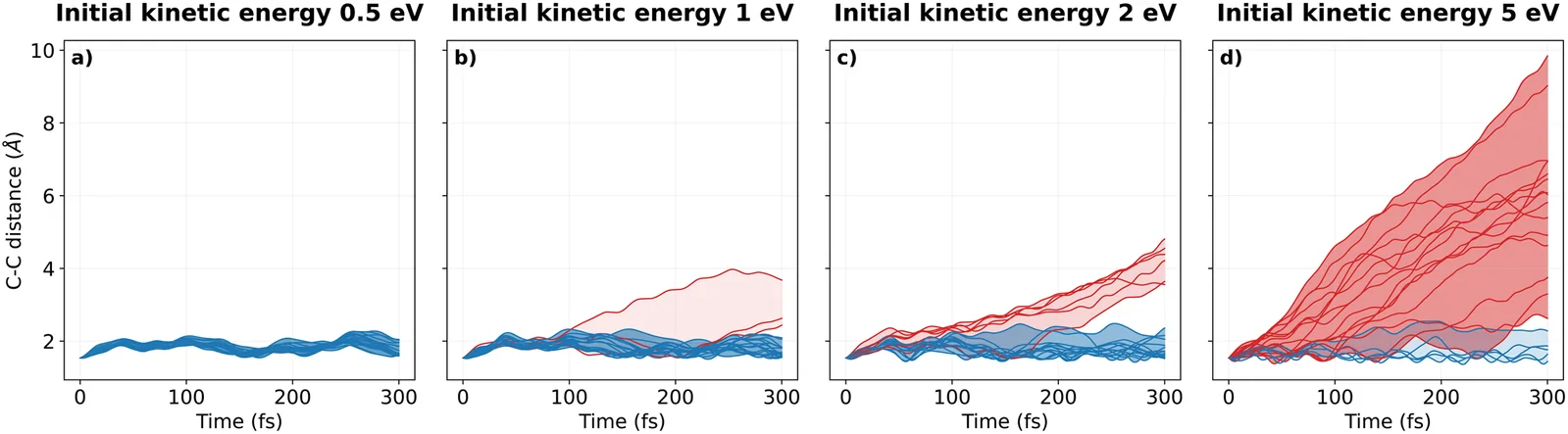
Evolution of C–C distances across 80 ADMP molecular dynamics trajectories. Trajectories are given for four initial nuclear kinetic energies, (a) 0.5, (b) 1, (c) 2, and (d) 5 eV, with 20 trajectories calculated for each energy. Red depicts the trajectories that undergo C–C bond fragmentation, while blue curves represent those that remain stable. The shaded regions indicate the boundaries of each group, with shading intensity set linearly proportional to the number of trajectories.

The choice of the second conformer is validated by a relaxed potential energy scan varying the C–C bond length, starting from its equilibrium geometry, as presented in Fig. 6. The initial geometry used is based on the equilibrium geometry of the second conformer of neutral glycine reported by Kasalová *et al.*^51^

**Fig. 6:**
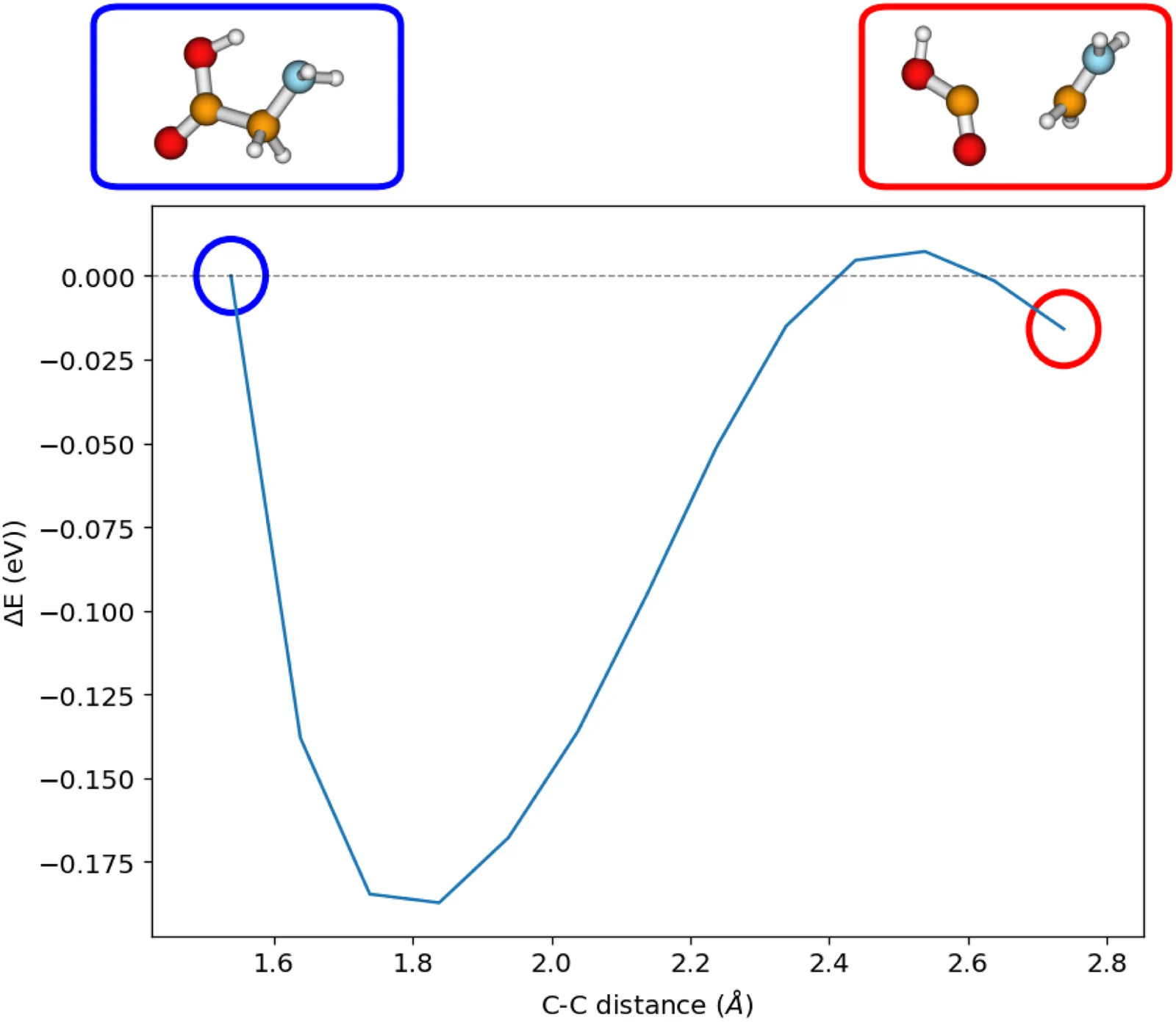
Energy profile along the relaxed scan of the C–C bond distance. Energies are in eV, and the optimized energy of the first geometry is set equal to zero. Calculations were done at the CAM-B3LYP/maug-cc-pVTZ level of theory, including Grimme's semiempirical dispersion with Becke–Johnson damping.

The energy profile in Fig. 6 indicates that while the initial C–C bond elongation upon ionization is barrierless, final cleavage into separate fragments requires overcoming a potential barrier. This corroborates that fragmentation requires excess energy to overcome the potential threshold, explaining the observed delay in our measurements. The final geometry corresponds to a C–C distance of 2.73 Å, where the resulting CH_2_=NH_2_^+^ fragment becomes planar with a carbon–nitrogen double bond characteristic of a stable iminium cation.

Extracting early-time dynamics (0–250 fs) would have allowed us to track the initial relaxation and the onset of fragmentation. As the generated cations are vibrationally hot, some fragments may emanate from vibrationally hotter states than others; the states of the cation and the final fragments would be broader and less separated, allowing us to better understand the underlying mechanism of fragmentation.

However, the laser-dressing effect introduces sidebands in photoelectrons, complicating the analysis and reducing confidence in the early-delay data. Alternatively, two X-ray pulses could be used to initiate and probe the dynamics. A pump pulse below the C 1s edge would yield valence and inner-valence ionization, which a second probe pulse could then track. This setup, available at the Maloja endstation and SwissFEL, provides sub-10 fs resolution without sideband artifacts. It would also enable site-selective excitation of the C 1s, N 1s, or O 1s edges, allowing us to observe how fragmentation varies with the initial ionization site.^29,53^

### State of the art

#### High laser intensity TR-XPS measurements

In addition to the cation and fragments photolines, the kinetic model incorporated the pre-*t*_0_ XPS spectrum and a no-laser reference spectrum. Both features were treated as a non-decaying step function in time. The pre-*t*_0_ spectrum represents the GSB and was assigned a fittable negative amplitude, while the no-laser spectrum was assigned a positive amplitude. Here, we discuss the underlying reasons for this, and why, if not accounted for, it can be misleading.

All electron spectrometers are sensitive to electrons originating from within a specific volume, dubbed the spectrometer's acceptance window. The acceptance window can be tuned to some extent in the case of hemispherical electron analyzers by changing the operation mode of the spectrometer and in the case of electron time-of-flight spectrometers by changing the extraction potential. The spectrometer acceptance window, if not optimized, can result in significant artifacts in time-resolved difference spectra, specifically in experiments that involve the pump-induced ionization of molecules in the gas phase. In such experiments, the effect of the excitation necessarily results in probed space-charge effects specifically within the ionization volume of the pump pulse, which may be smaller than the acceptance window of the spectrometer (Fig. 7c). These heterogeneous space-charge effects occur precisely in the same region as the pump–probe signal, including the GSB, and accordingly give rise to artifacts in the TR-XPS difference spectra in the GSB region.

**Fig. 7:**
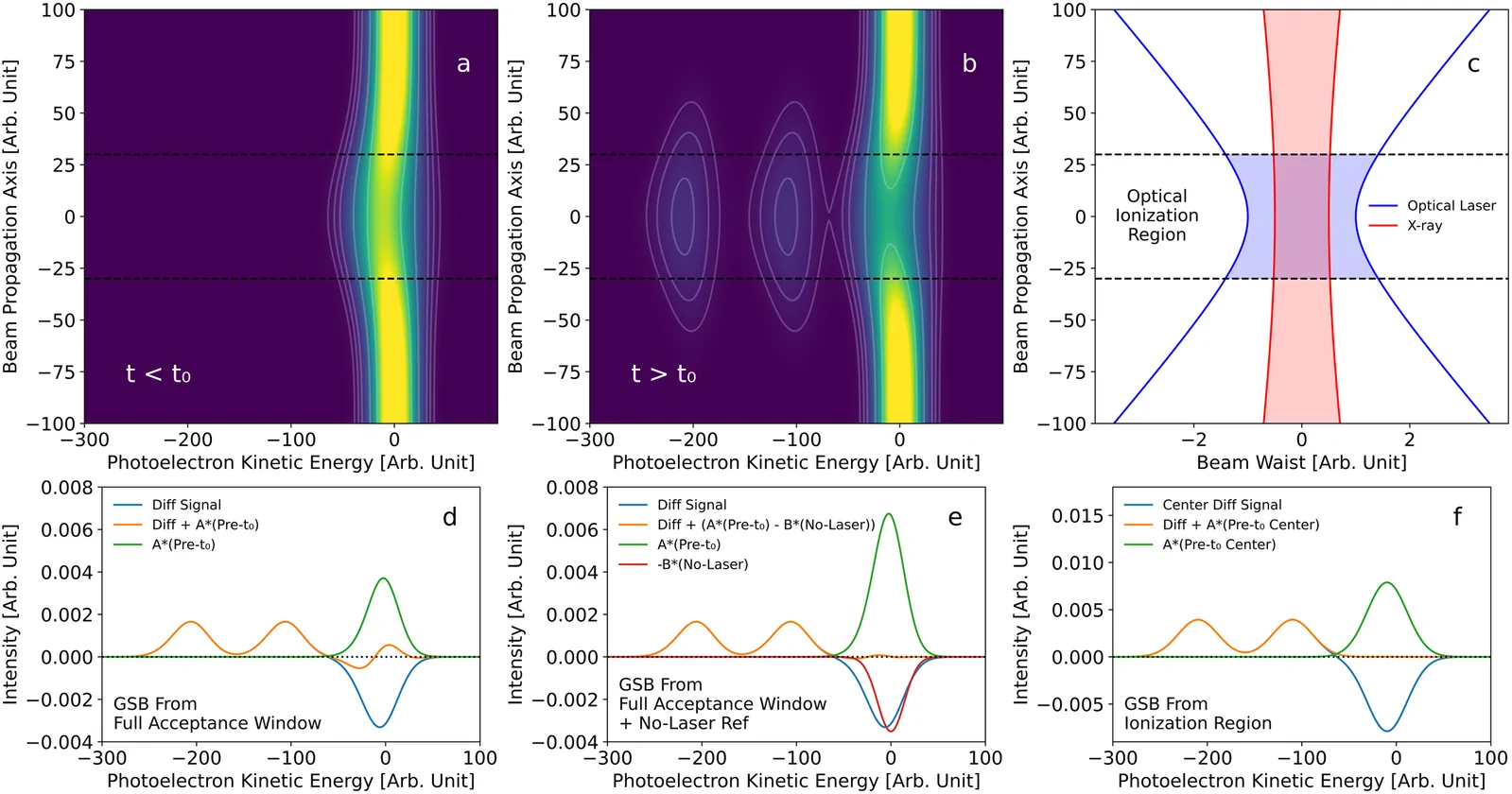
(a and b) Simulated 1s photoline before (a) and after (b) photoexcitation, as a function of the beam propagation axis through the spectrometer acceptance window. (c) Gaussian beam waist of the optical (pump) beam and the X-ray (probe) beam showing the pump ionization region (dotted lines). (d–f) Simulated difference spectra and fits of the ground-state bleach component taking projections along the entire acceptance window (d), the same but with an added unmodulated component (e), and taking projections only within the ionization region (f).

To investigate the origin of this artifact, we simulated the photoline along the beam propagation axis of the X-ray and laser beams including space-charge effects within the Rayleigh range of the optical laser. Fig. 7a shows the simulated photoline pre-*t*_0_ as measured by the hemispherical electron analyzer, while Fig. 7b shows the same post-*t*_0_ and includes a pump–probe signal in addition to the space-charge effect. The space-charge effect is simulated as a modulation in the width and position of the photoline through the focus (and thus, ionization region) of the pump beam. The difference spectra in Fig. 7d and e are generated by taking the projections of the pre-*t*_0_ and post-*t*_0_ signal along the entire acceptance window of the spectrometer and subtracting them from each other. In these cases, the GSB component is taken as the projection of the pre-*t*_0_ signal, as is common practice in time-resolved spectroscopy experiments. When attempting to fit the GSB component, it is apparent that a residual with a differential shape is always present in the bleach region and cannot be removed by adjusting the intensity of the GSB component (Fig. 7d, orange curve). This can easily lead to the misinterpretation that a separate positive transient spectral feature with a very small chemical shift (a hot ground state) exists underneath the bleach and acts to counteract it. However, this artifact exists only because the pre-*t*_0_ spectrum, which includes a mixture of photoelectrons that feel and photoelectrons that do not feel space-charge effects, is in fact a poor representation of the true GSB spectrum which only includes photoelectrons that have been modulated by space-charge effects. This is confirmed in Fig. 7e, where the fit residual can be greatly reduced by adding back a component of the photoline that does not include space-charge effects (Fig. 7e, red curve). Finally, in Fig. 7f, the same is done with projections from only the ionization region within the acceptance window. In this case, the pre-*t*_0_ signal is a perfect representation of the GSB spectrum, and no other components are necessary to fit the bleach component of the difference signal. Fig. 7 shows this effect manifesting in the presented data on glycine.

These findings demonstrate that matching the spectrometer's acceptance window to the laser excitation region is critical for TR-XPS measurements. If the acceptance window is larger than the excitation volume, space-charge artifacts can mimic immediate hot ground state formation, manifesting as a broad positive feature. These artifacts can be mitigated by including a laser-free spectral reference in the fit model. Alternatively, when using a hemispherical electron analyzer in imaging mode, area dispersed detection can be used to select a region-of-interest confined to the excited volume.

#### Sidebands and time resolution

The cross-correlation between the pump and probe pulses limits the time resolution in a pump–probe experiment, manifesting as signal smearing during the temporal overlap. Using high-intensity beams can introduce nonlinear artifacts that completely obscure the short-time-scale signals. In optical transient absorption spectroscopy, for example, cross-phase modulation introduces signal artifacts that are independent of the sample response. Such artifacts may dominate the signal on timescales longer than the expected cross-correlation.

In the presence of a strong laser field, the photoelectron can absorb *n* photons of the driving field, shifting its kinetic energy by *n* quanta of the optical photon energy. The C 1s map of glycine (Fig. 8a, top) shows this effect clearly over a 500 fs window around *t*_0_, despite measured pump and probe durations of 30 fs and 70 fs, respectively. The sidebands' temporal and spectral structures are well resolved, a testament to the high-resolution spectral and temporal resolution of the measurement. This phenomenon arises because the laser field strongly dresses the photoelectrons, driving multi-photon exchanges even at the leading and trailing wings of the pulse. In such experiments, time resolution is limited not by the FWHM durations but by the observable sideband window.

**Fig. 8:**
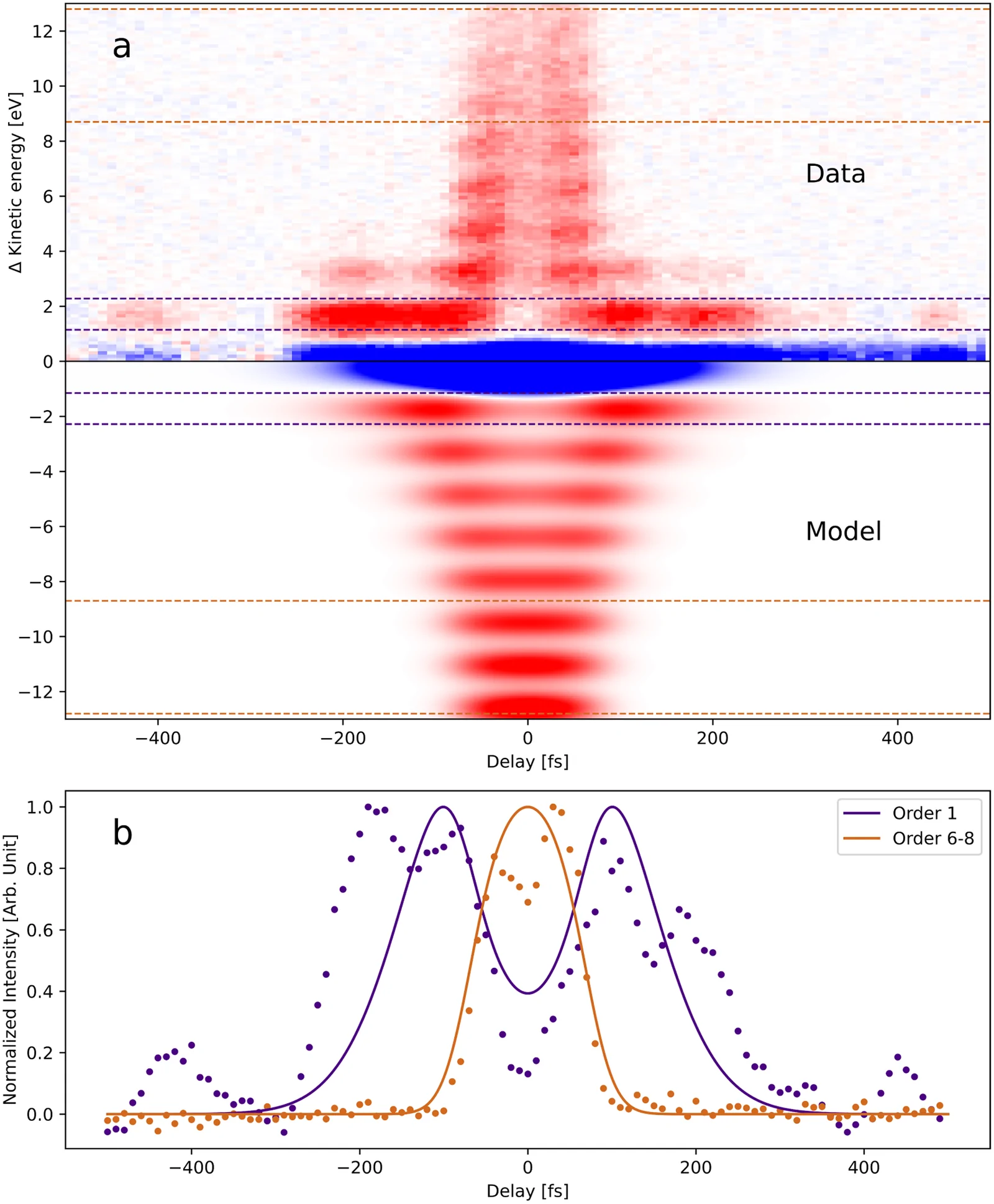
(a) Glycine C 1s sideband data (top) and model (bottom). (b) Normalized transients of the 1st order (purple) and 6–8th order sidebands.

As is the case with the cross-phase modulation artifact in optical transient absorption spectroscopy, if the artifact can be understood then it can be modeled and its effect potentially removed from the data. Towards this end, a simple model based on ref. 54 was constructed to reproduce the observed sidebands in the presented data. This model is based on the continuum coupling of a free electron with the optical dressing field and calculates the amplitudes (and depletion) of *n* sidebands by acting on the free-electron plane wave with raising and lowering operators, which act to raise or lower the kinetic energy of the electron by *n* times the optical photon energy. It takes as parameters the X-ray and optical photon energies, the core-ionization potential of the atomic site, the X-ray and optical pulse durations and their intensity ratio. Fig. 8a, bottom, shows the result of the model for the case of a glycine C 1s photoelectron within an 800 nm dressing field. The characteristic narrowing of the sideband temporal profile with increasing Δ*E* is reproduced as well as the depletion of the *n* = 0 (unstreaked photoline) and *n* = 1 sidebands, as depicted in Fig. 8b. Currently, the model assumes Gaussian X-ray and laser pulse profiles, which may be an important shortcoming as the weak wings of the laser pulses often deviate from a perfect Gaussian, and the laser pulse may include pre- or post-pulses, which can also contribute to fully temporally-separated sidebands, as is also observed in the presented glycine data (see Fig. 8b and 2a–c, *t* = 430 fs).

While such a model may help subtract the ground-state sideband contribution, the situation is only partially remedied. These sidebands appear across all spectra within the temporal overlap, including the signals of the cation, fragments, or, in other cases, electronically excited states. This results in significant spectral congestion and complex interference. Removing this artifact requires including the sidebands in the fittable model in the global routine, thereby increasing fitting complexity considerably. For experiments requiring extreme time resolution and high intensities, this will be necessary. On the other hand, in experiments with low laser intensities, the number of sidebands is reduced to 1 or 2, making it easier to fit.

#### Difficulties in theory/calculations

To simulate TR-XPS of molecular systems of chemical interest, three main issues must be properly addressed:

• Reliable modeling of the interaction between the pump pulse and the molecular system;

• Accurate simulation of nuclear dynamics triggered by the pump pulse;

• Accurate modelization of transient XPS signature.

In this study, we focus on Gly^+^ fragmentation at delays exceeding 200 fs. We prioritize the computational characterization of intermediates and products over high-precision nuclear dynamics. Accordingly, the theoretical focus remains on the modelization of the transient XPS signature.

The simulation follows a two-step approach:

• In the first one, intermediates and products of Gly^+^ fragmentation were identified with simulations based on the ADMP molecular dynamics model. Although this model relies on a classical and adiabatic description of the nuclear motions, it proved to be reliable to identify fragmentation intermediates and products of the rovibrationally hot Gly^+^ produced by the pump pulse;

• In the second one, transient XPS signatures were modeled, collecting *ab initio* calculations of static XPS signals for several representative nuclear configurations of Gly^+^ and its main fragmentation products.

The main issue behind reliable modeling of TR-XPS signatures is the need for a protocol that can simulate XPS signatures across different nuclear configurations, especially when these configurations are associated with a substantial chemical change (*e.g.*, a bond cleavage).

The first difficulty concerns the number of core-ionized electronic states that must be simulated to reproduce experimentally observed XPS signatures. In many cases, the most intense XPS signature is not due to a transition to the lowest core-ionized state, forcing scientists to include in the computational protocol an ensemble of several core-ionized states in order to reproduce at least the main XPS signature. As consequence, computational resources required to calculate core-ionized electronic states are larger than those needed to calculate the ground electronic state or the lowest excited electronic states. In other words, calculation of core-ionized states is the computational bottleneck in the modeling of TR-XPS signatures: therefore, the selection of a large number of core-ionized electronic states greatly increases the computational resources (and time) needed to perform calculation; at the same time, the inclusion of a small number of core-ionized states in the computational protocol could leads to misleading and incorrect results.

The choice of the active space is also critical: here, the main issue is the choice of an active space suitable to reliably simulate different electronic states at different nuclear configurations, *i.e.*, before and after a bond cleavage (*e.g.*, the C–C bond cleavage experimentally observed in the present study). Orbital rotations may introduce discontinuities in the calculated TR-XPS signatures.

A pivotal point is the choice of the active space for the initial geometry (in this case, the Gly^+^ at the equilibrium geometry of conformer I of neutral glycine). This active space can then be tested on the fragmentation product of interest (in this case, COOH and CH_2_NH_2_^+^). Then, to reproduce the smooth dependence of a TR-XPS signature on time (*i.e.*, on different nuclear configurations, in this case mainly the C–C bond length), the active space should be re-optimized at each subsequent nuclear configuration, starting from the active space of the previous one. In other words, the idea is to sequentially optimize the active space for all the nuclear configurations of interest {***R***_*k*_} (with *k* = 1, …, *N*) following the sequence *R*_1_ → *R*_2_ → *R*_3_ → ⋯ → *R*_*N*_. This approach is successful if two conditions are met: (i) the differences between two subsequent nuclear configurations *R*_*n*_ and *R*_*n*+1_ are small enough to prevent abrupt changes in the orbital space during the electronic optimization and (ii) the computational cost of a single XPS calculation is small enough to allow a sequential calculation of the XPS signatures of different nuclear configurations, which will be gathered to construct the final TR-XPS signal. A pictorial representation of the computational workflow is presented in Fig. 9.

**Fig. 9:**
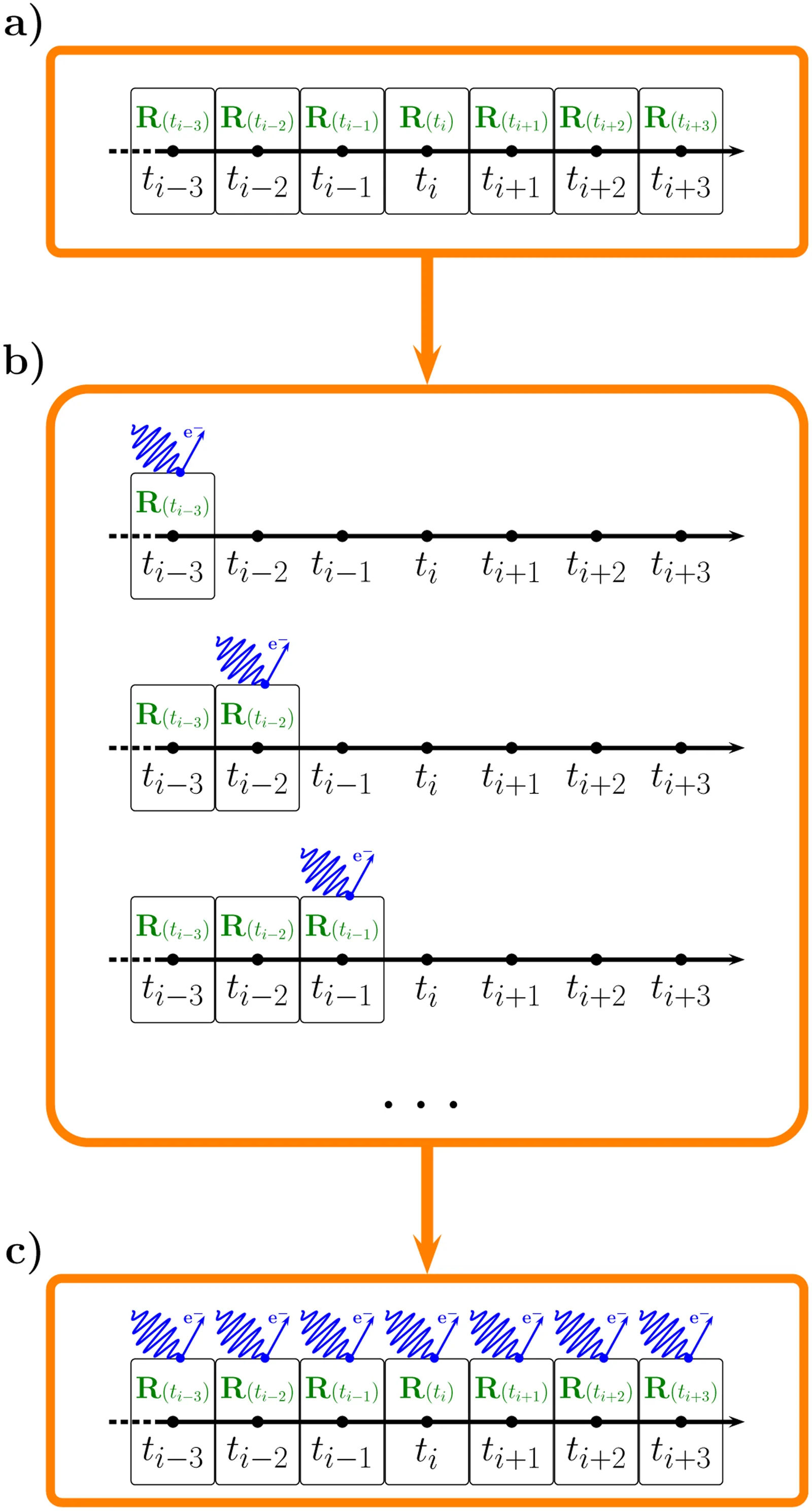
Pictorial representation of a workflow to simulate TR-XPS spectra. (a) A set {***R***_*k*_} of relevant nuclear configurations is selected: the set can be obtained through systematic alteration of a relevant degree of freedom (*e.g.*, the elongation of the C–C bond of Gly^+^), or directly computed (*e.g.*, with a suitable *ab initio* molecular dynamics approach). (b) Static XPS signatures are calculated for each nuclear configuration *R*_*k*_, starting the MOs optimization from the set of MOs optimized for the previous nuclear configuration *R*_*k*−1_ (to prevent undesired orbital rotations). This step is the computational bottleneck of the computational protocol, because (i) XPS calculations are performed sequentially and (ii) calculation of core-ionized electronic states is needed. (c) Static XPS signatures calculated at the previous step are collected, giving the final TR-XPS spectrum (XPS signatures as function of time).

Other potential sources of error (*e.g.*, the use of Dyson Amplitudes to determine the intensity of TR-XPS signatures) are expected to be less relevant. However, they could play a role in some circumstances.

Finally, the ensemble size is determined by testing trial sets (*e.g.*, 5, 10, 20, 30, and 40 states) to choose the smallest set reproducing all relevant signatures. However, this may fail if the required number of core-ionized states is not enough to reproduce the XPS signatures as the nuclear configuration change.

## Conclusions

We employ TR-XPS to monitor the dynamics of Gly^+^ in real time by probing the chemical environment at the C 1s, N 1s, and O 1s edges. Unlike traditional mass spectrometry or ion-coincidence techniques, which typically measure asymptotic yields and branching ratios, TR-XPS enables instantaneous probing of local chemical environments during dissociation. Supported by ADMP simulations and post Hartree–Fock calculations, we analyze the transition maps and identify a Gly^+^ lifetime of (395 ± 25) fs, corresponding to the C–C bond cleavage into neutral COOH and positively charged CH_2_NH_2_^+^. The Gly^+^ lifetime indicates that dissociation is driven by nuclear vibrational motion rather than by electronic degrees of freedom. The barrier was confirmed by the trajectories showing vibrationally “hot” cations overcoming a potential energy barrier.

By probing all the constituent elements in the molecule, we have constructed a detailed picture of the fragmentation dynamics with high sensitivity to the local charge states of individual fragments.

In addition, we discuss critical artifacts in TR-XPS, including space-charge artifacts, artifacts that can mimic hot ground state signatures, and limitations on observing the earliest dynamics due to the presence of sidebands. We also address theoretical challenges regarding active-space stability in core-ionized systems. Ultimately, this work demonstrates the unique power of site-selective TR-XPS to resolve local electronic environments and track the structural evolution of complex molecules in real time, and will help guide future TR-XPS studies.

## Author contributions

Antoine Sarracini: methodology, investigation, formal analysis, visualization, writing – original draft. Lorenzo Paoloni: conceptualization, methodology, formal analysis, visualization, writing – original draft. Chris Arrell: data curation. Sven Augustin: data curation. Loïc Bassement: data curation, writing – review and editing. Christoph Bostedt: data curation, writing – review and editing. Sergio Díaz-Tendero: conceptualization, writing – original draft. Davide Faccialá: data curation, writing – review and editing. Zhaoheng Guo: data curation. Gregor Knopp: data curation. Suddhasattwa Mandal: data curation, writing – review and editing. Ana Sofia Morillo Candas: data curation. Kevin Prince: data curation, writing – review and editing. Zhibin Sun: data curation. Hankai Zhang: data curation. Xinhua Xie: methodology, investigation, data curation, writing – review and editing. Antonio Picón: conceptualization, methodology, supervision, formal analysis, writing – original draft. Kirsten Schnorr: methodology, investigation, data curation, writing – original draft. Andre Al Haddad: methodology, investigation, formal analysis, validation, writing – original draft, supervision, project administration.

## Conflicts of interest

There are no conflicts to declare.

## Supplementary Material

FD-OLF-D6FD00069J-s001

## Data Availability

The data required to reproduce the results are publicly available at the DOI https://doi.org/10.5281/zenodo.20699793 on the Zenodo server. While only the reduced data is uploaded, access to the raw measured data is granted upon request. Supplementary information (SI) is available. See DOI: https://doi.org/10.1039/d6fd00069j.
