# Supplementary material for: Time-resolved core-level photoelectron spectroscopy of glycine fragmentation: a case study on the capabilities and limitations of site-selective probes at XFELs
Source: Faraday Discuss. 2026 Jun 17. Online ahead of print. doi: 10.1039/d6fd00069j (PMC13660752; doi:10.1039/d6fd00069j)
Supplement: Supplementary file 1 [file FD-OLF-D6FD00069J-s001.pdf]

Supplementary Information (SI) for Faraday Discussions.
This journal is © The Royal Society of Chemistry 2026

Electronic Supplementary Information

Time-Resolved Core-Level Photoelectron Spectroscopy of Glycine Fragmentation: A Case Study on the Capabilities and Limitations of Site-Selective Probes at XFELS

S1 Energy Calibration

The XPS energy axes were calibrated by recording the 1s photoline of a molecule in the gas phase with a known binding energy. CO2 (binding energy 297.7 eV [Myrseth *et al.*(2002)Myrseth, Bozek, Kukk, Saethre, and Thomas]) was used for the C1s data and N2O was used for the N1s (binding energy 408.5 eV) and O1s (binding energy 541.4 eV) data[Hedin *et al.*(2014)Hedin, Tashiro, Linusson, Eland, Ehara, Ueda, Zhaunerchyk, Karlsson, Pernestål, and Feif The calibration procedure is outlined in Fig. S1. The photoline of the reference gas is recorded for at a series of hemisphere kinetic energies and the peak positions are fit to a line to retrieve the eV/pixels which is used to generate the kinetic energy axis. Finally the kinetic energy axis is subtracted from the photon energy to obtain the binding energy axis. Fig. S1c shows the result of the calibration for the CO2 reference gas measurements.

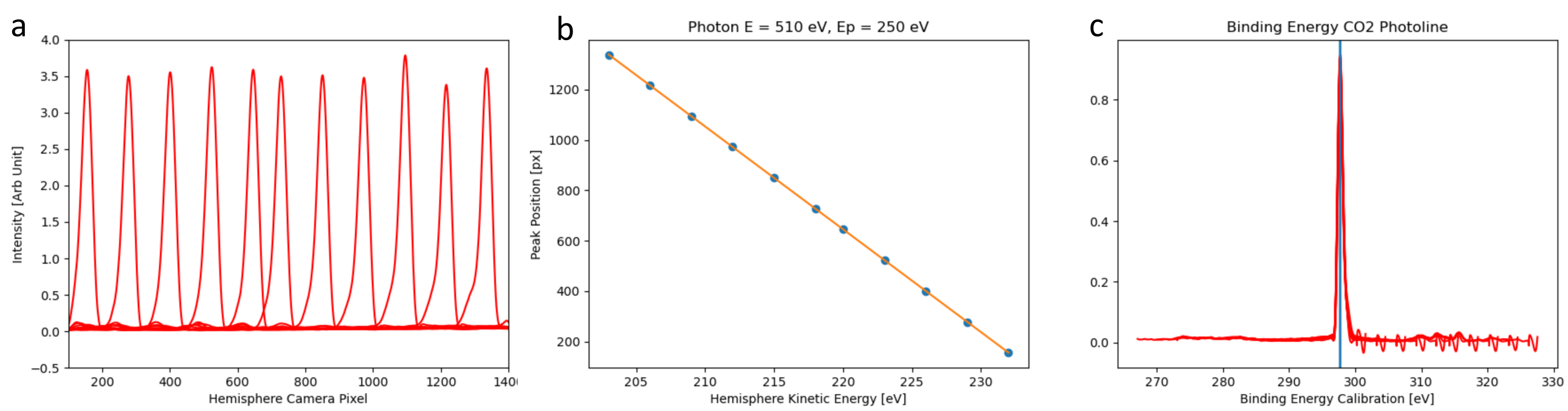

Figure S1: (a) Measured C1s photoline of CO2 for a series of hemispherical electron analyzer kinetic energies. b) Fit of peak position in pixels vs. kinetic energy. c) Calibrated binding energy axes for CO2 reference spectra.

S2 Model residuals and fit parameters

- For each element, add the residual map and parameters, including error bars.

After the model is optimized, a projection of the data and model are overlayed for comparison. Fig. S3 shows the projection of the model vs the data for spectra after 500 fs. The residual clearly shows that the model captures all the spectral features. A non variant peak at 420 eV, appears in the data, but could not be attributed to Glycine. The main peaks F1 and F2 belong to the Glycine cation and CH2NH2+, respectively.

To ensure that the model captures both the transient dynamics and the spectral features, we take transient cuts at chosen binding energies. Figure S4 shows transients taken at the peaks of the GSB in green, cation in red, fragment in purple, and brown for the background peak. The transients from the model and measurements are overlaid, and the residuals are plotted under them. The transients clearly show that we are not missing any temporal components, especially for the cation lifetime. The residual is large at timescales close to time zero because the sidebands overshadow the glycine signal. The model was initialized with physical values as described in the main text, using observations and theoretical guidance. The fitting procedure starts with global optimization using the Nelder-Mead method, interrupted sporadically by the LBFGSB method to introduce

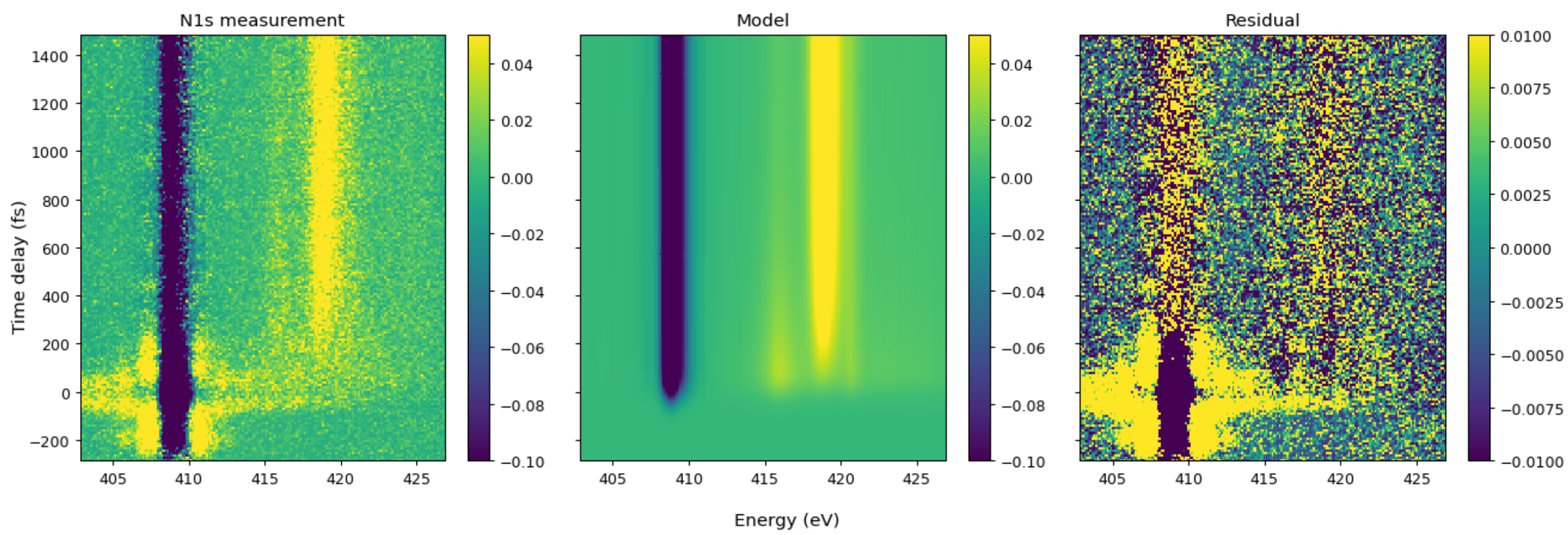

Figure S2: TR-XPS N1s measurement (left), fitted model (center) and residuals (right). The residuals after fitting are structureless in time and energy outside of the intense sideband signal region.

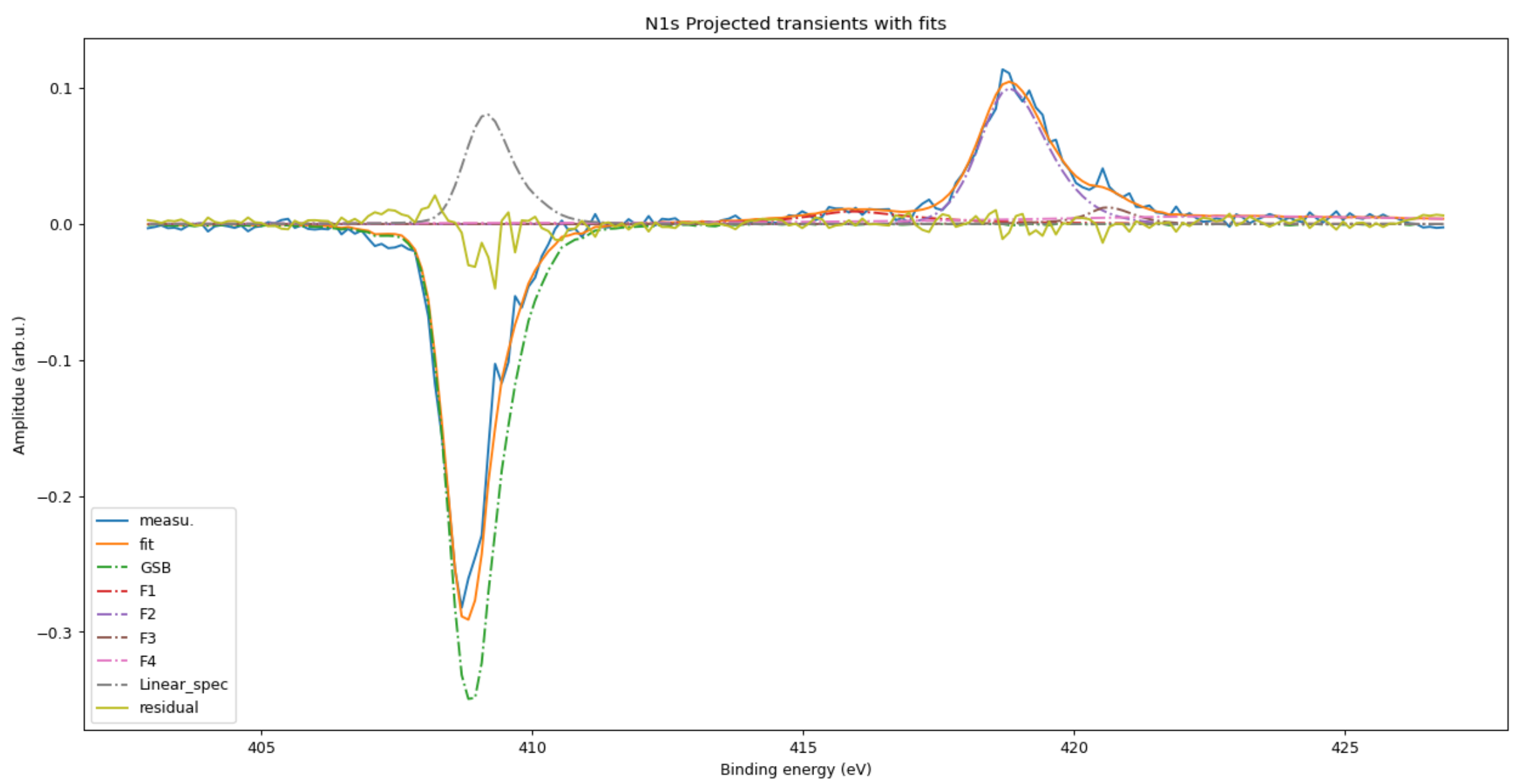

Figure S3: N1s XPS difference signal (blue) and fit (orange) integrated after $t = 500$ fs showing the spectral decomposition (dashed lines) into the GSB (green), "no laser" (grey) and product components (other colors) along with the fit residuals (yellow).

randomness, all within the LMFIT Python package [LMF()]. The parameter boundaries were set reasonably, as depicted in the figure below.

Global optimization was performed using stochastic methods (Nelder–Mead and lbfgsb) over 100,000 iterations to ensure thorough exploration of parameter space and to avoid convergence to local minima. The resulting parameter set was subsequently refined using a local least-squares optimization to obtain a consistent solution and to evaluate parameter uncertainties.

Due to the high dimensionality of the model and the presence of correlated parameters, conventional covariance-based uncertainty estimates were found to be unreliable for the full parameter set. Therefore, confidence intervals were evaluated selectively for a subset of physically relevant and well-identifiable parameters, while less sensitive or strongly correlated parameters were held fixed during the analysis. This approach improves the stability of the local curvature estimation required for confidence interval determination.

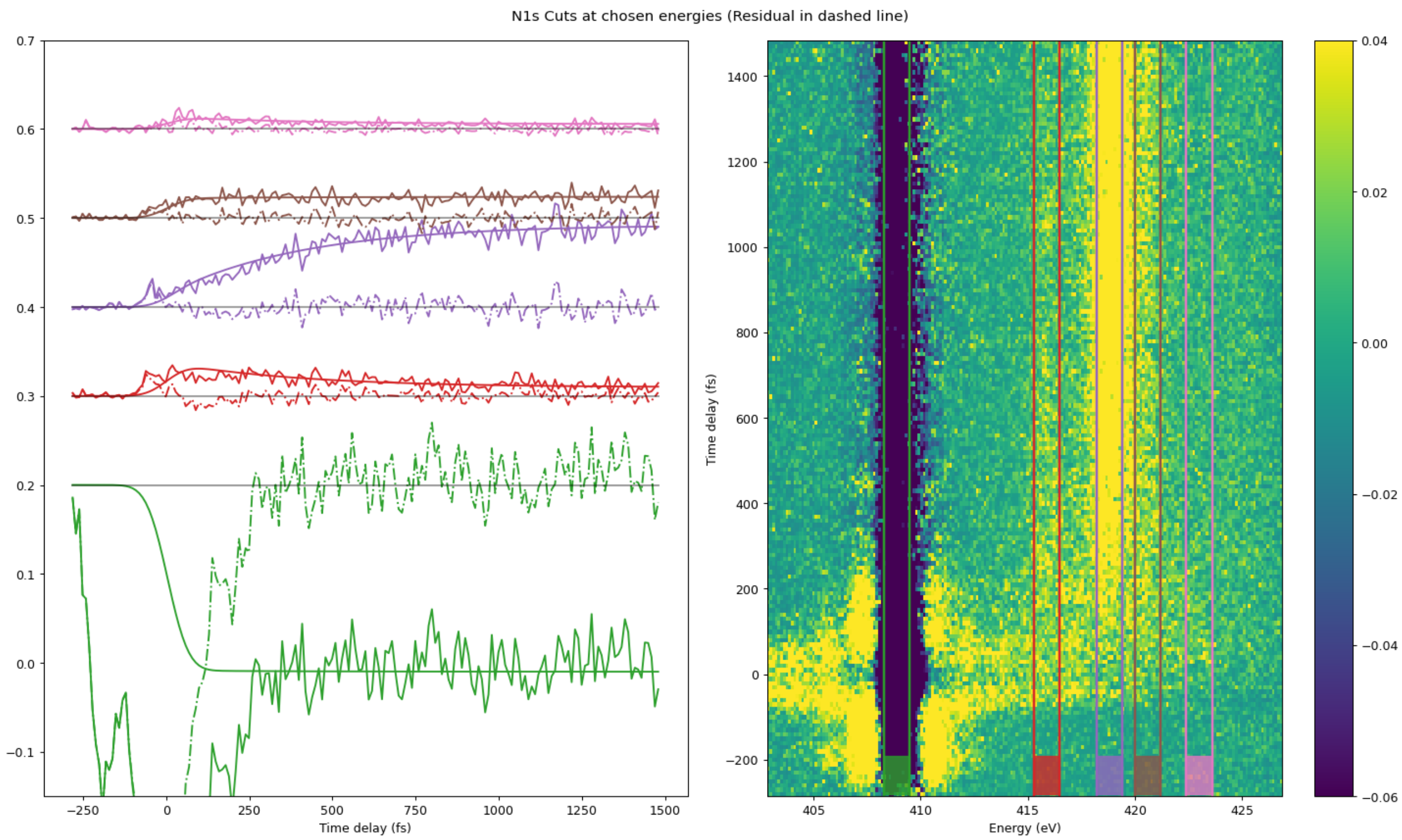

Figure S4: Integrated transients of the N1s TR-XPS measurement, global fit (solid) and residuals (dashed) (left) along with their corresponding regions of interest overlaid on the map (right).

To ensure robust estimation of confidence intervals, parameter bounds were chosen sufficiently broad to avoid artificially constraining the solution while still remaining within physically meaningful limits. Confidence intervals were determined for key observables, including the spectral widths and amplitudes of the cation and fragment species as shown in Fig. S6, as well as the cation lifetime, which governs both its decay and the rise dynamics of the fragment. Additionally, global parameters affecting all species were included in the uncertainty analysis.

Similarly, we present the fits and residuals for the C1s transient maps. We present the data, model, and residual side by side in Fig. S7. The residual shows fluctuations around the GSB, which could be due to laser fluctuations during the acquisition of this dataset. Note that the residual map colormap is set 10x lower than the model and measurements, hence enhancing the artifacts.

Next, we present the projection after 600 fs, showing the fit, the residuals, and the individual calculated transitions. The starting states were determined based on data observations and close guidance from the theory, as described in the main text.

Finally, the transients are also presented at chosen binding energies. Despite the congested spectra, and red shifting peak, we do obtain an excellent fit. Note, that the time scale for the cation was used a global parameters from the N1s data, which was optimized to 395 fs.

S3 Inclusion of the no laser spectrum

Here, we present the fit procedure for the ground-state bleach of N1s with and without the "no laser" reference spectrum. It is clear from the residual that the essential need is to add this spectrum. In addition, the residual when no laser spectrum is added can be misleading and is considered a shifted excited spectrum.

Parameter Name	Value	Std Error	Vary	Min	Max
GSB_E	-4.2726e-03	None	True	-1.00e+01	1.00e+01
GSB_Sigma	1.6245e-01	None	True	0.00e+00	3.00e+01
GSB_C	3.5525e-01	None	True	0.00e+00	1.10e+01
GSB_A0	1.3143e+00	None	True	0.00e+00	1.50e+01
F1_E	4.1590e+02	None	True	4.15e+02	4.17e+02
F1_Sigma	1.9377e+00	None	True	7.00e-01	2.00e+00
F1_C	1.2225e-01	None	True	1.00e-02	1.40e+01
F1_A0	1.8535e+00	None	True	0.00e+00	1.50e+01
F1_A1	6.1051e+00	None	True	0.00e+00	1.50e+01
F1_assymtriclineshape_a	3.6350e-01	None	True	5.00e-02	5.00e-01
F1_assymtriclineshape_b	6.1997e-01	None	True	1.00e-01	8.00e-01
F1_assymtriclineshape_gl	1.0000e+00	None	True	0.00e+00	1.00e+00
F2_E	4.1880e+02	None	True	4.18e+02	4.20e+02
F2_Sigma	1.3345e+00	None	True	7.00e-01	2.00e+00
F2_C	1.4541e+00	None	True	1.00e-02	1.40e+01
F2_A0	2.5445e-05	None	True	0.00e+00	1.50e+01
F2_A1	9.4588e-01	None	True	0.00e+00	1.50e+01
F2_assymtriclineshape_a	1.6947e-01	None	True	5.00e-02	5.00e-01
F2_assymtriclineshape_b	5.3653e-01	None	True	1.00e-01	8.00e-01
F2_assymtriclineshape_gl	4.6497e-01	None	True	0.00e+00	1.00e+00
Linear_spec_E	4.5412e-03	None	True	-1.00e+00	1.00e+00
Linear_spec_Sigma	8.9622e-01	None	True	0.00e+00	5.00e+00
Linear_spec_C	1.0000e+00	None	True	9.99e-01	1.00e+00
Linear_spec_A0	8.2857e-02	None	True	0.00e+00	5.00e+00
Tau_T0	8.7485e-04	None	True	0.00e+00	1.00e-03
Tau_Tinf	1.0000e+06	None	True	1.00e+06	1.00e+06
Tau_TF11	3.9453e+02	None	True	1.00e+02	1.00e+04

Figure S5: N1s fit parameters and their optimized values.

--- Confidence Interval Report ---

	99.73%	95.45%	68.27%	_BEST_	68.27%	95.45%	99.73%
F1_Sigma:	-0.27449	-0.15754	-0.04313	2.00000	+inf	+inf	+inf
F1_C :	-inf	-inf	-inf	0.21430	+0.00634	+0.01268	+0.01902
F1_A0 :	-inf	-inf	-inf	0.91067	+0.04871	+0.09742	+0.14614
F1_A1 :	-inf	-inf	-inf	3.31362	+0.18959	+0.37919	+0.56880
F2_Sigma:	-0.03111	-0.02087	-0.01050	1.31571	+0.01062	+0.02137	+0.04237
F2_C :	-1.23555	-1.23323	-1.23140	1.32579	+0.00849	+0.01698	+0.02548
F2_A0 :	-inf	-inf	-inf	0.00000	+0.00398	+inf	+inf
F2_A1 :	-inf	-inf	-inf	1.07970	+0.00692	+0.01383	+0.02075
Tau_TF11:	-24.26864	-16.26266	-8.17483	394.53933	+8.25926	+16.60797	+25.04873

Figure S6: Relevant N1s parameter confidence intervals obtained from a least squares fitting loop performed using the optimized parameters retrieved from the global fitting routine.

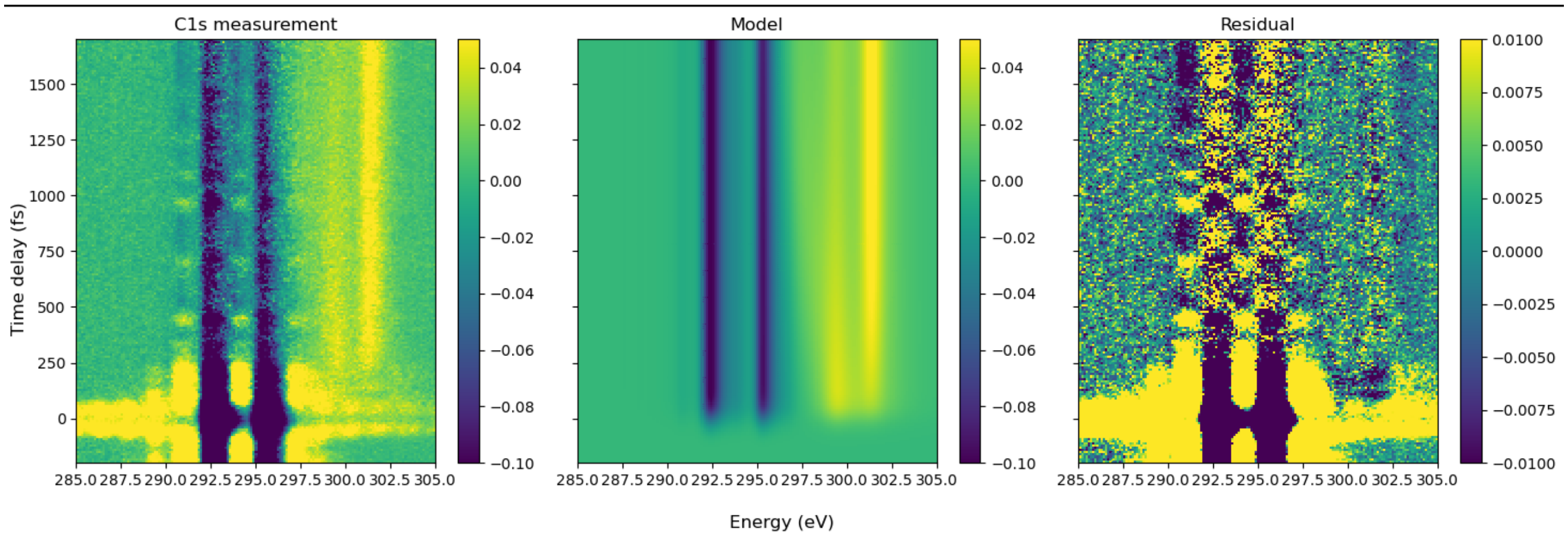

Figure S7: TR-XPS C1s measurement (left), fitted model (center) and residuals (right). The residuals after fitting are structureless in time and energy outside of the intense sideband signal region.

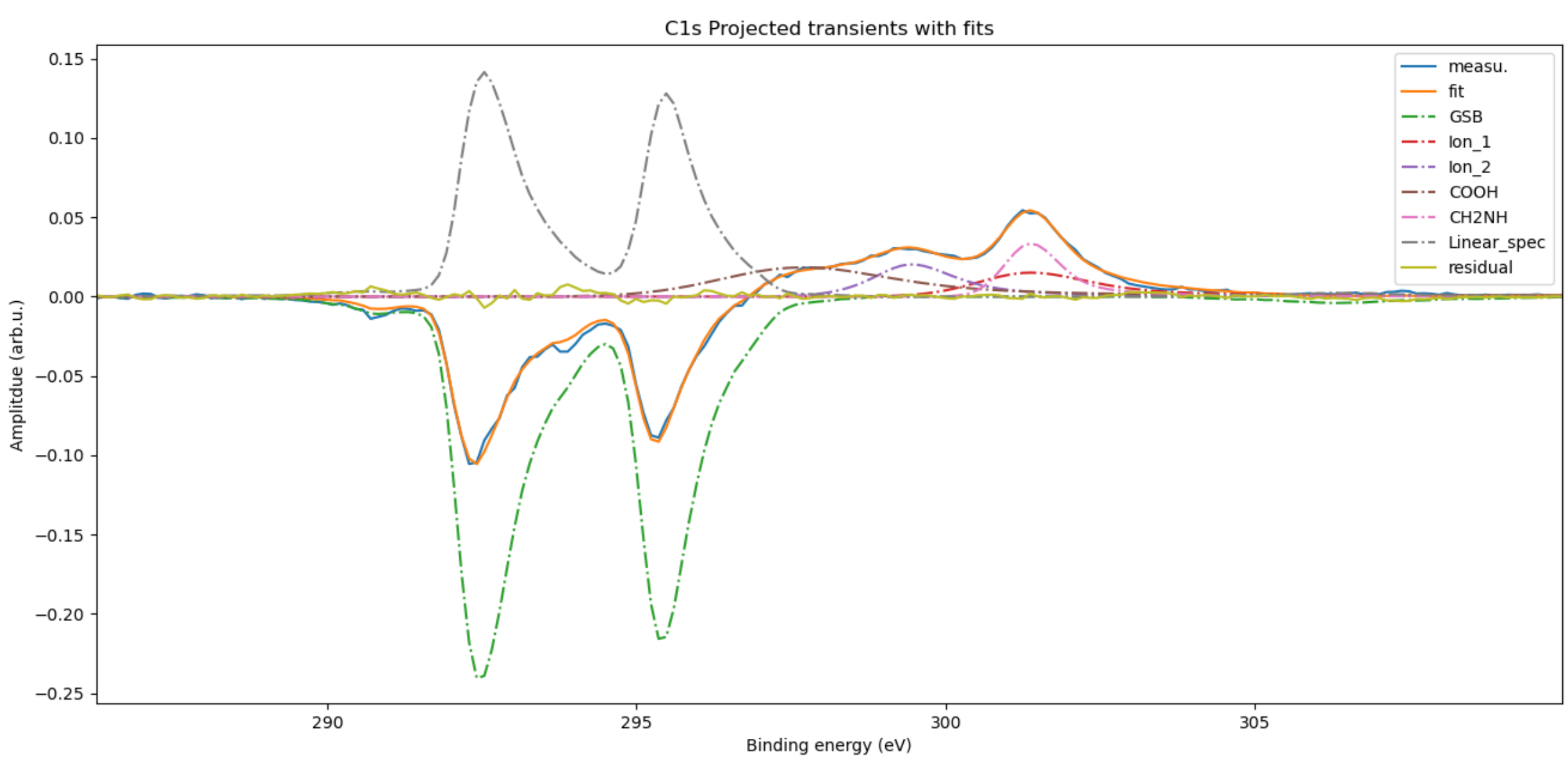

Figure S8: C1s XPS difference signal (blue) and fit (orange) integrated after $t = 600$ fs showing the spectral decomposition (dashed lines) into the GSB (green), "no laser" (grey) and product components (other colors) along with the fit residuals (yellow).

S4 Stick model of Glycine neutral conformers

The initial geometry is the equilibrium geometry of the five conformers of neutral glycine. The 2 most stable conformers of neutral glycine were experimentally observed in free jet expansion experiments [Kasalová *et al.*(2007)Kasalová, Alle

S5 Static XPS spectra at carbon, nitrogen and oxygen K-edge calculated for the 5 most stable neutral glycine conformers

Static XPS were calculated for the 5 most stable conformers of neutral glycine molecule. Results are shown in figure S12. In the main text, only the calculated XPS of the most stable conformer of neutral glycine is reported

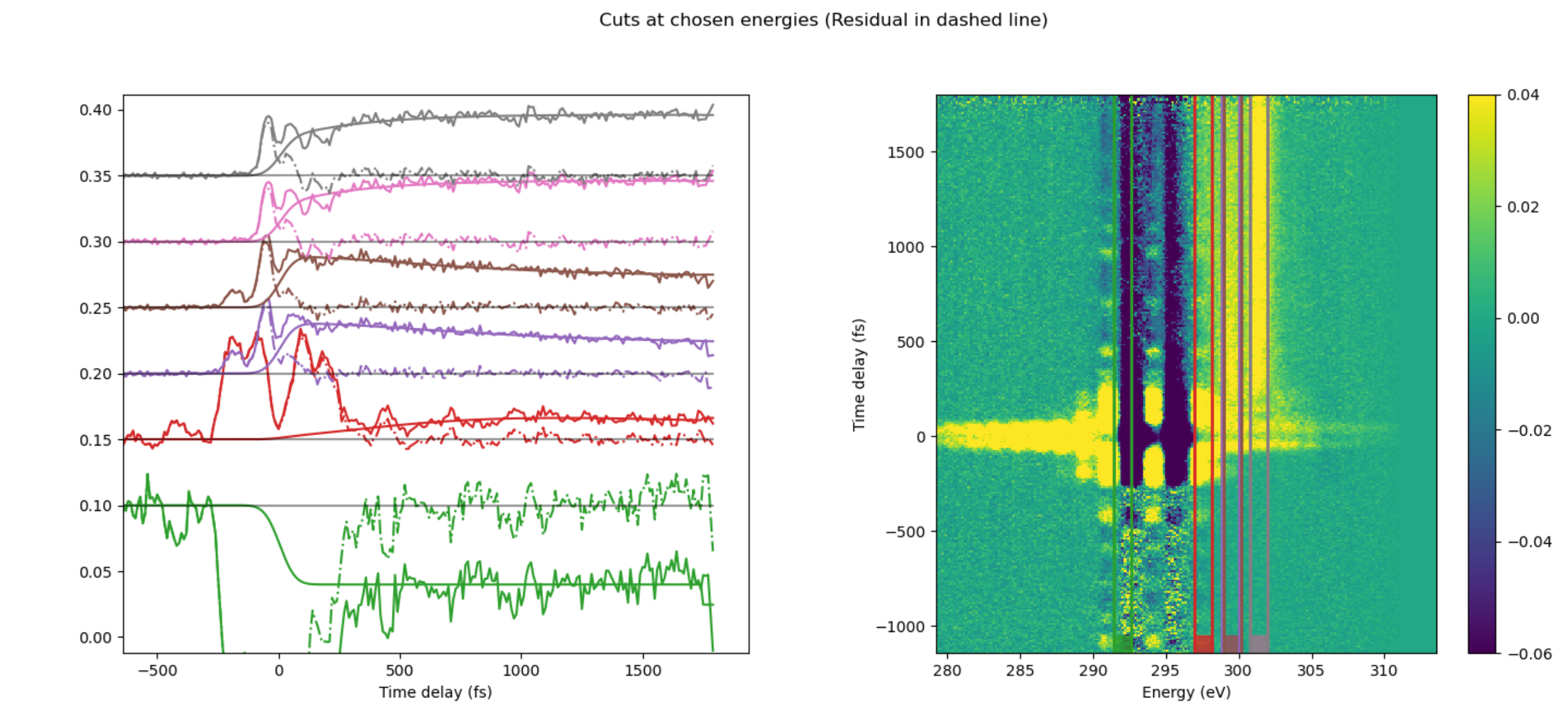

Figure S9: Integrated transients of the C1s TR-XPS measurement, global fit (solid) and residuals (dashed) (left) along with their corresponding regions of interest overlaid on the map (right).

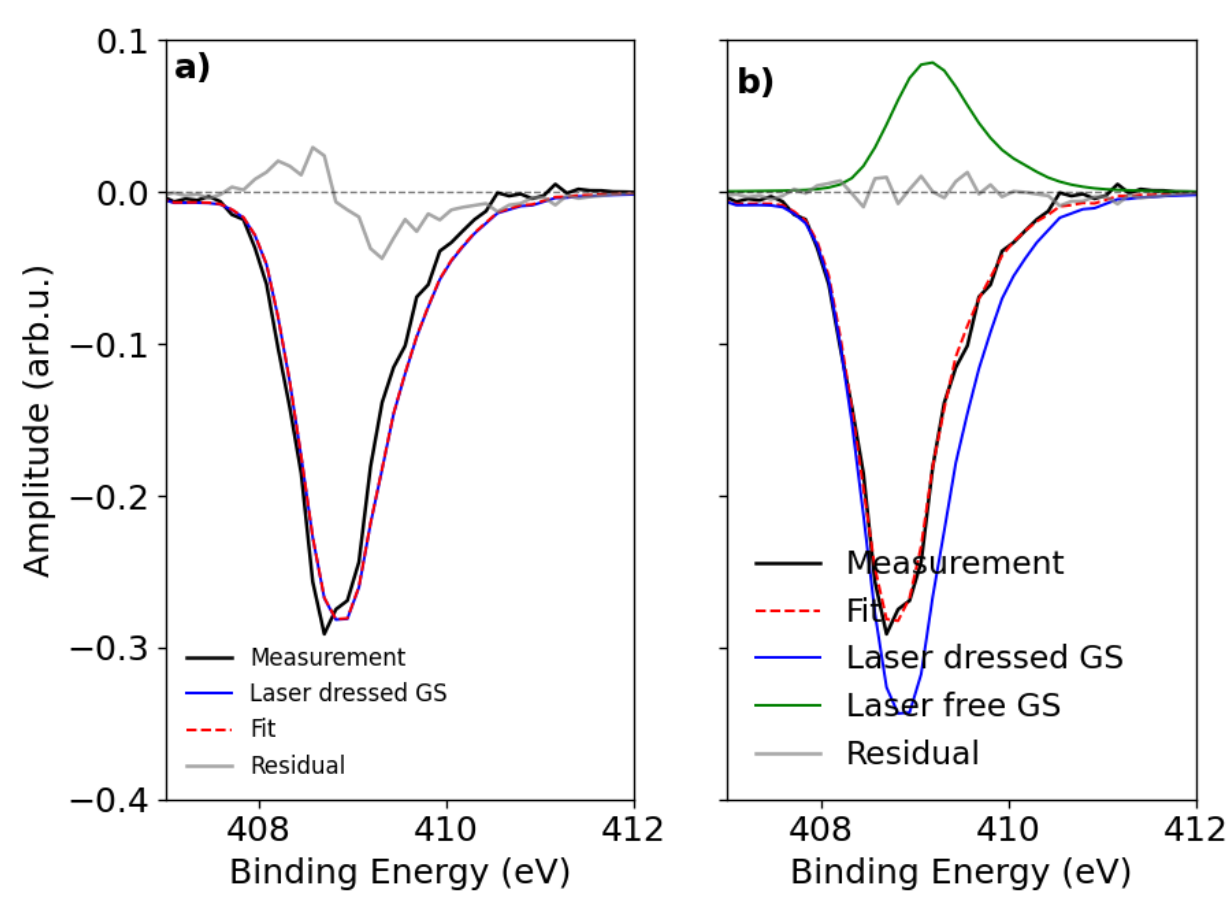

Figure S10: a) The fit of Glycine N1s GSB taking only the pre-t_0 as the GSB spectrum and (b) showing the same plus "no-laser" component, mitigating the effect to within the signal-to-noise baseline.

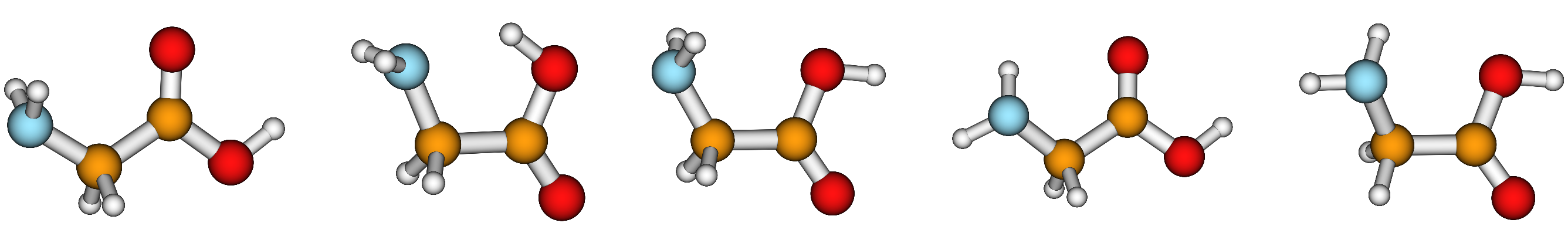

Figure S11: The 5 most stable conformers of the neutral glycine molecule. Conformers 1-5 are laid from left to right as reported by Kasalova et al.

for comparisons with experimental XPS data.

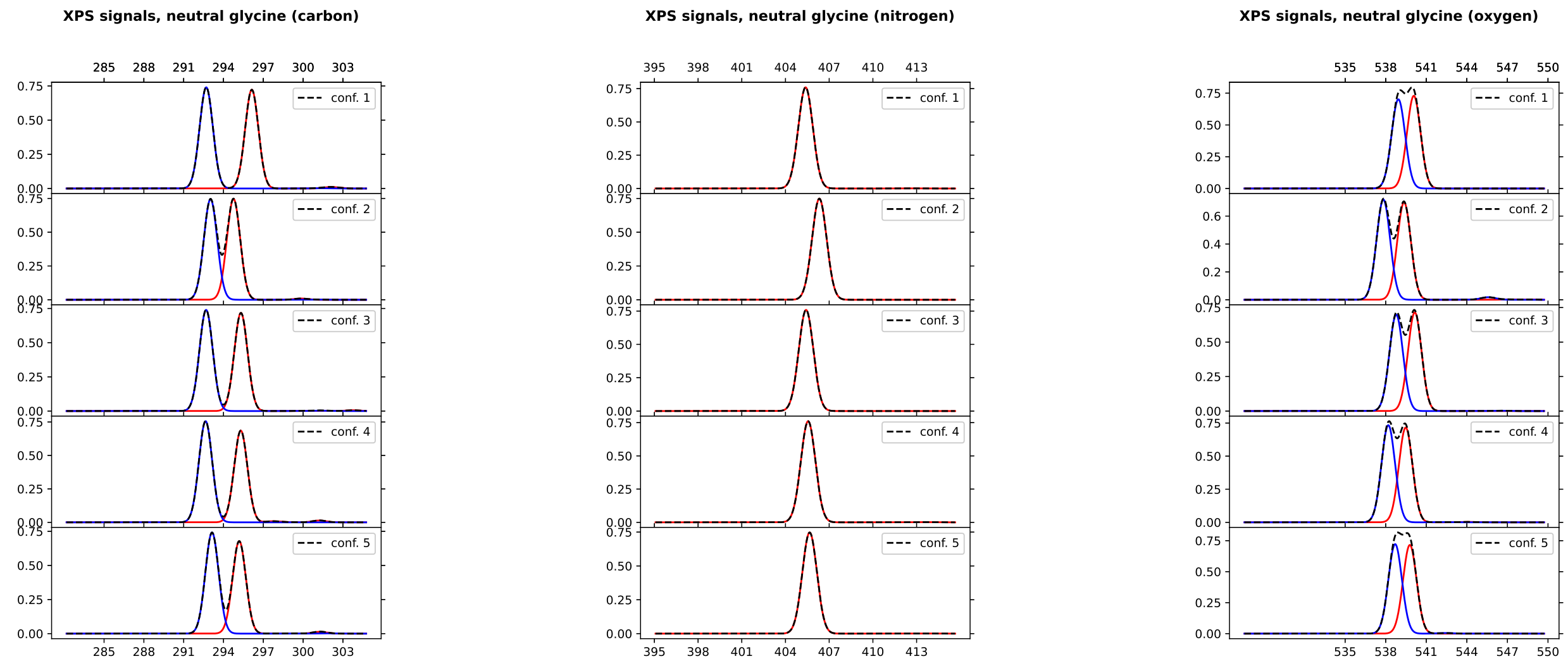

Figure S12: Calculated static XPS (carbon, nitrogen, and oxygen K-edges) of the 5 most stable conformers of neutral glycine.

S6 Elongation of the C-C bond of glycine cation: relaxed scans starting from the 2 most stable conformers of neutral glycine

In this section, we report the results of the relaxed scan performed for the elongation of the C-C bond. Only the results obtained for the second most stable conformer are reported in the main manuscript. In the case of the most stable conformer, an energy barrier is observed when the C-C bond is elongated. Anyway, the discontinuity observed for small values of the C-C distance clearly suggest that some other structural element (i.e., something different from the C-C bond distance) substantially affects the potential energy surface of the cationic ground state.

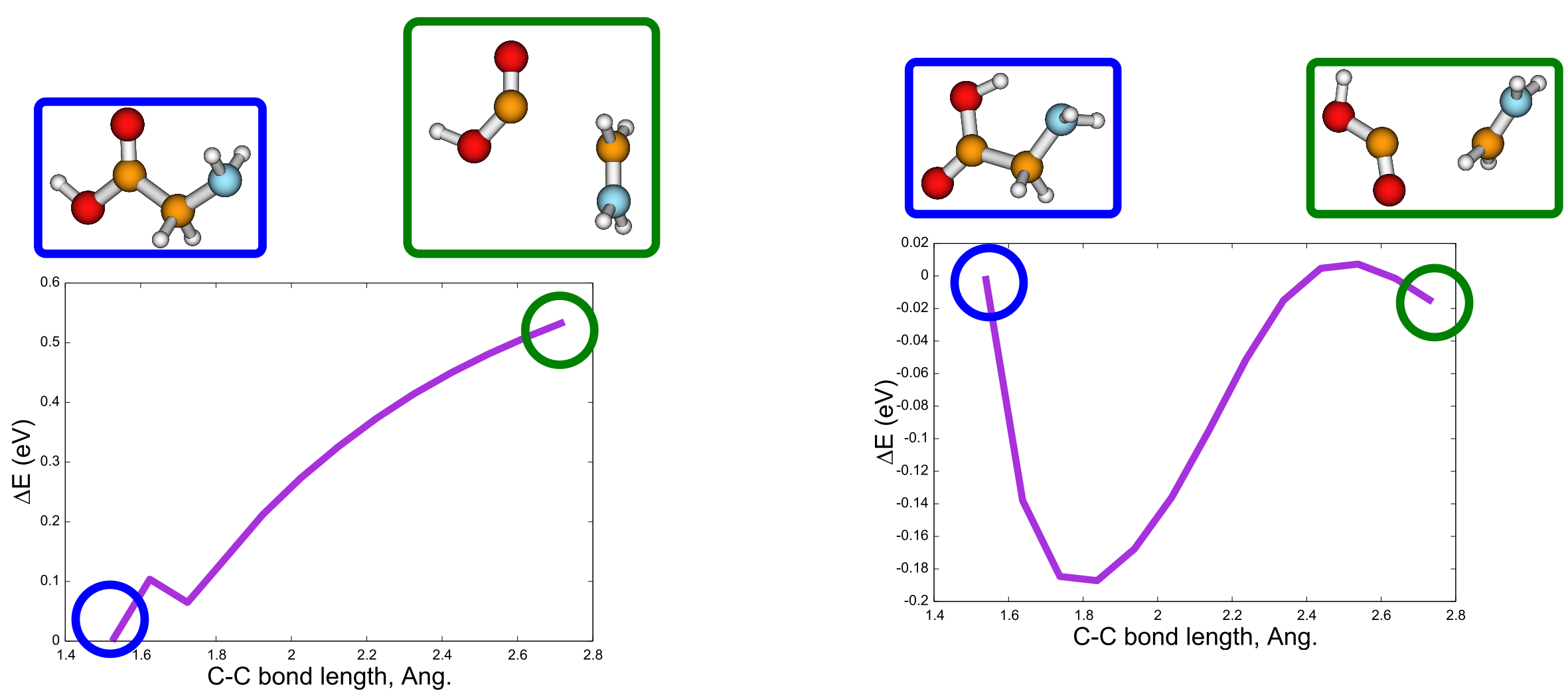

Figure S13: Relaxed scan for the elongation of the C-C bond length of glycine cation. The initial geometries correspond to the 2 most stable conformers of neutral glycine.

References

[Myrseth *et al.*(2002)Myrseth, Bozek, Kukk, Saethre, and Thomas] V. Myrseth, J. D. Bozek, E. Kukk, L. J. Saethre and T. D. Thomas, *J. Electron Spectrosc. Relat. Phenom.*, 2002, **122**, 57–63.

[Hedin *et al.*(2014)Hedin, Tashiro, Linusson, Eland, Ehara, Ueda, Zhaunerchyk, Karlsson, Pernestål, and Feifel] L. Hedin, M. Tashiro, P. Linusson, J. H. D. Eland, M. Ehara, K. Ueda, V. Zhaunerchyk, L. Karlsson, K. Pernestål and R. Feifel, *J. Chem. Phys.*, 2014, **140**, 044309.

[LMF()] *Performing Fits and Analyzing Outputs*, `https://lmfit.github.io/lmfit-py/fitting.html`, Accessed: 2026-06-3.

[Kasalová *et al.*(2007)Kasalová, Allen, Schaefer III, Czinki, and Császár] V. Kasalová, W. D. Allen, H. F. Schaefer III, E. Czinki and A. G. Császár, *Journal of Computational Chemistry*, 2007, **28**, 1373–1383.
